# Economic burden of cardiovascular disease in the United Kingdom

**DOI:** 10.1093/ehjqcco/qcaf011

**Published:** 2025-02-24

**Authors:** Kingsley Shih, Naomi Herz, Aziz Sheikh, Ciaran O'Neill, Paul Carter, Michael Anderson

**Affiliations:** Department of Emergency Medicine, University Health Network, Toronto, ON M5G 2C4, Canada; British Heart Foundation, London NW1 7AW, UK; Nuffield Department of Primary Care Health Sciences, University of Oxford, Oxford OX2 6GG, UK; Usher Institute, College of Medicine and Veterinary Medicine, The University of Edinburgh, Edinburgh EH16 4UX, UK; Centre for Public Health, School of Medicine, Dentistry and Biomedical Sciences, Queen's University Belfast, Belfast BT12 6BA, UK; Victor Phillip Dahdaleh Heart and Lung Research Institute, University of Cambridge, Cambridge CB2 0BB, UK; Centre for Primary Care Health Services research, University of Manchester, Oxford Rd, Manchester M13 9PL, UK; LSE Health, Department of Health Policy, London School of Economics and Political Science, London WC2A 2AE, UK

**Keywords:** Cost of illness, Cardiovascular disease, Economic burden

## Abstract

**Background and aims:**

Direct (medical and non-medical) and indirect (production losses and informal care) costs of cardiovascular disease (CVD) have been captured in two previous United Kingdom (UK) cost-of-illness studies, but the areas of long-term care and medical device costs were neglected. We aimed to quantify the economic burden of CVD in the UK from a societal perspective between the fiscal years 2019/20 and 2021/22.

**Methods and results:**

Mixed-methods study in a prevalence-based retrospective review of economic costs focused on the public sector. Top-down costing was applied to the following areas: inpatient hospital care, outpatient specialist care, emergency care, primary care, medications, medical devices, long-term care, production losses to morbidity, and production losses to mortality. Bottom-up costing was used by applying the marginal effects of having a CVD on several parameters using survey data from the Survey on Health, Aging, and Retirement in Europe to estimate informal care costs. The modelling performed shows that the total costs of CVD in the UK in 2021/22 were £29.021 billion (bn), with direct costs of £16.620 bn and indirect costs of £12.402 bn. The breakdown of direct costs for the UK were inpatient care (£6.732 bn), long-term care (£4.649 bn), medications (£1.940 bn), primary care (£1.556 bn), outpatient care (£1.011 bn), emergency care [£327.6 million (mn)], and medical devices (£404.4 mn). The breakdown of indirect costs for the UK were informal care costs (£6.377 bn), production losses to mortality (£4.544 bn), and production losses to morbidity (£1.481 bn).

**Conclusion:**

There is a significant economic burden of CVD in the UK, with the highest direct cost resulting from inpatient care and the highest indirect cost resulting from informal care.

## Introduction

Cardiovascular disease (CVD) has long been one of the major causes of morbidity and mortality globally.^1^ As population's age and multi-morbidity increases, the burden of non-communicable diseases (NCDs) such as CVD will only continue to place increasing strain on resource-limited health systems. Currently, CVD already accounts for the greatest proportion of NCD-related deaths globally.^1^ In the United Kingdom (UK), CVD accounts for two of the top five causes of death (i.e. ischaemic heart disease and cerebrovascular disease) and is the second most common cause of dementia.^2^

As we move forward in addressing and preventing the effects of CVD, we must understand the economic impact posed by CVD relative to other medical conditions. By quantifying the societal impact of a certain disease or class of diseases in economic terms, we are not only able to determine and compare the impact across diseases, but also build a case for intervention programs, the allocation of research funding, forecast clinical and preventative needs, and provide the cost aspect to economic program evaluation.^3^ Understanding costs of NCDs across sectors also provides insights into which services are most impacted to inform service delivery and planning.

There have been several studies of the cost of illness (COI) of CVD in the UK over the past three decades. Previous studies have taken a societal perspective (i.e. capturing both direct medical costs and indirect costs such as productivity loss due to morbidity and mortality), though the methodologies and scope of the studies have varied.^4,5^ The core components of inpatient hospital care, outpatient specialist care, primary care appointments, emergency care, medications, productivity losses due to morbidity and mortality, and informal care costs are included in previous studies.^4,5^ However, there are some cost components that have been neglected.

The most recent full assessment of COI was published in 2017 by Wilkins *et al.* as a part of *European Cardiovascular Disease Statistics 2017*, using 2015 data sources, which found a total cost of £19.5 billion (bn) (£22.14 bn in 2021).^5^ This study took a societal perspective, however, it did not include social and community care within its scope. The next study in recency was published in 2006 by Luengo-Fernandez *et al.* using data from 2004, finding that the total cost was £29.1 bn (£40.02 bn in 2021). This study did include social and community care costs, though costs of medical devices were not included.

The inclusion of long-term care (LTC) in the calculation of CVD-related COI is an integral component because CVD comprises the two most common diagnoses of individuals in residential care—dementia and stroke. A previous study has estimated that dementia [of which 17% can be attributed to vascular dementia (VaD)] comprises 43% of all nursing home residents in Belgium, while stroke comprises 36% of all individuals admitted to nursing homes because of a medical condition.^6,7^ It is estimated that the UK government spends £50.49 bn on nursing and residential care, amounting to 22% of the total health and care expenditure in 2022.^8^

Further to this, previous studies have not included the cost of medical devices and advanced treatments. Over the past decade, the scope of investigations and treatments available in the cardiovascular space has widened with new technological developments. A good example of this is the advent of endovascular thrombectomy for the treatment of large-vessel ischaemic strokes. This treatment can be life-changing, but also depends on advanced imaging techniques, specialized clinical expertise, and intravascular devices to achieve good outcomes.^9,10^ Currently, National Health Service (NHS) England spends £549 million on high-cost medical devices and goods, which are not included in the Healthcare Resource Groups (HRG) that are currently paid to providers for their management of patients; of this amount, CVD-related devices make up 44.5% of this expenditure as per the NHS England Schedule of Costs.^11^

This study aimed to quantify the economic burden of CVD in the UK from a societal perspective between the fiscal years 2019/20 and 2021/22, including important drivers of costs (i.e. medical devices and LTC) not incorporated in previous studies. The 2021/22 fiscal year has the most contemporaneous data available due to publication delays, and the 2019/20 fiscal year represents the last pre-Coronavirus-19 (COVID-19) year. These costs are compared with the figures generated using 2015 data in the European Heart Network (EHN) study.

## Methods

This study was carried out using a mixed-methods approach of top-down costing and bottom-up costing taking a societal perspective. We focused solely on healthcare costs for the public sector, as there is no reliable data on privately funded care for cardiovascular care in the UK. Moreover, the private sector currently accounts for a relatively small proportion of healthcare spending in the UK.^12^ Our methods conform with guidelines for COI studies published by Schnitzler *et al.* 2023.^13^ Costing was performed for each of the four nations within the UK. The analysis was undertaken for the fiscal years 2019/2020, 2020/21, and 2021/22 as the 2021/22 year is the most recent year where data are published in all four nations, while the 2019/20 year was included as it is the last pre-COVID-19 pandemic year. CVD is defined in the International Classification of Diseases, 10th edition (ICD-10) as categories I00-I99. Although this list is expansive, it is not exhaustive. The British Heart Foundation has expanded this definition to include other vascular conditions, including VaD, which have similar underlying risk-factors to the diseases that fall within the traditional ICD-10 classification.^14,15^ There were 10 cost components estimated in this COI study, with five of them being modelled costs (primary care, LTC, production losses to morbidity, production losses to mortality, and informal care costs). The remaining five components were costs taken directly from publicly available NHS data sources [inpatient hospital care, outpatient specialist care, accident & emergency (A&E) care, medications, and medical devices]. Intangible healthcare costs were not included, specifically those associated with reduced quality of life associated with CVD.^16^ All analysis was carried out on Excel 16.75.2 for Mac, and Stata 17 SE.

### Data sources

All data sources used to estimate direct and indirect costs are summarized in *Table 1*, broken down by the four nations of the UK.

**Table 1:** Summary of data sources used for the collection and analysis of cost data, by Nation

Cost component	Description	Reference
***Direct costs***		
Inpatient care		
England	§ NHS England 2019/20 National Cost Collection	NHS Digital^17^
Scotland	§ Scottish Health Service—CostsBook 2019/20	Public Health Scotland^18^
Wales	§ NHS Wales Expenditure Programme Budgets 2019/20 § NHS Wales Programme Budgets Quality Report	StatsWales^19^ StatsWales^20^
Northern Ireland	§ Northern Ireland HRG Unit Costs Schedules 2019/20	NHS Northern Ireland^21^
Outpatient care		
England	§ NHS England 2019/20 National Cost Collection	NHS Digital^17^
Scotland	§ Scottish Health Service—CostsBook 2019/20	Public Health Scotland^18^
Wales	§ NHS Wales Expenditure Programme Budgets 2019/20 § NHS Wales Programme Budgets Quality Report	StatsWales^19^ StatsWales^20^
Northern Ireland	§ NHS NI Outpatient First and Follow-up Appointments	NHS Northern Ireland^22^
Accident & emergency care		
England	§ NHS England 2019/20 National Cost Collection	NHS Digital^17^
Scotland	§ Scottish Health Service—CostsBook 2019/20	Public Health Scotland^18^
Wales	§ Number of attendances in NHS Wales emergency departments by age band, sex and site § NHS Wales Programme Budgets 2019/20	NHS Wales^23^ StatsWales^19^
Northern Ireland	§ Hospital statistics: emergency care activity 2019/20	NHS Northern Ireland^24^
Primary care		
England	§ Unit Costs of Health and Social Care 2022 Manual § Appointments in General Practice § Impact of changes to national guidelines on hypertension-related workload: an interrupted time series analysis in English primary care	Curtis & Burns (2019)^25^ NHS Digital^26^ Lay-Flurrie *et al.* (2021)^27^
Scotland	§ Scottish Health Service—Costsbook 2019/20 § Impact of changes to national guidelines on hypertension-related workload: an interrupted time series analysis in English primary care	Public Health Scotland^18^ Lay-Flurrie *et al.* (2021)^27^
Wales	§ NHS Wales Expenditure Programme Budgets 2019/20 § Impact of changes to national guidelines on hypertension-related workload: an interrupted time series analysis in English primary care	StatsWales^19^ Lay-Flurrie *et al.* (2021)^27^
Northern Ireland	§ NHS Northern Ireland General Medical Services Provision § Impact of changes to national guidelines on hypertension-related workload: an interrupted time series analysis in English primary care	NHS Northern Ireland^28^ Lay-Flurrie *et al.* (2021)^27^
Medications		
England	§ NHS England Prescription Cost Analysis 2019/20	NHSBSA^29^
Scotland	§ NHS Scotland Prescription Cost Analysis 2019/20	Public Health Scotland^30^
Wales	§ NHS Wales Prescribing Cost Analysis 2019/20	GOV.WALES^31^
Northern Ireland	§ Prescription Cost Analysis for Northern Ireland 2020	GOV.UK^32^
Long-term care		
England Scotland Wales Northern Ireland	§ Sentinel Stroke Audit Programme—National Clinical Audit § Projections of older people with dementia and costs of dementia care in the United Kingdom, 2019–2040 § Social Care 360: Expenditure	SSNAP UK^33^ Wittenberg *et al.* (2019)^34^ King's Fund^35^
Medical devices		
England Scotland Wales Northern Ireland	§ NHS England 2019/20 National Cost Collection § Estimates of the population for the UK, England, Wales, Scotland, and Northern Ireland	NHS Digital^17^ Office for National Statistics^36^
***Indirect costs***		
Production loss: morbidity		
England Scotland Wales Northern Ireland	§ Disability Living Allowance: Cases in Payment—Data from May 2018 § Employment and Support Allowance—Data from May 2018 § Sickness absence in the UK labour market § Employee earnings in the UK	Department for Work and Pensions^37,38^ Office for National Statistics^39,40^
Production loss: mortality		
England Scotland Wales Northern Ireland	§ Mortality Statistics—underlying cause, sex, and age § Labour Market Overview, UK § Earnings and Hours Worked, age group: ASHE Table 6	Official Census & Labour Market Statistics^41^ Office for National Statistics^42,43^
Informal care		
England Scotland Wales Northern Ireland	§ Labour Market Overview, UK § Estimates of the population for the UK, England, Wales, Scotland, and Northern Ireland § Survey of Health, Aging, and Retirement in Europe, Waves 1–8	Office for National Statistics^36,42^ Borsch-Supan *et al.* (2022)^44^^–52^

### Direct costs

#### Inpatient hospital care

Inpatient costs were estimated through the summation of CVD-related HRG currency codes that hospital providers are paid by (see *Table 1*—Inpatient care). These codes encompass the entire cost of clinical care for diagnoses and are stratified by the complexity of the patient and their stay, and thus provide an accurate picture of CVD-related hospital costs, within the limitations of the data collected. This information was available for England, Scotland, and Northern Ireland. The currency codes used for extraction, any modifications, and their descriptions can be found in online Appendix A. For General Medicine admissions, a proportion was attributed to CVD as per a pan-European survey on General Medicine case-mix, which found that 17.9% of admissions were CVD-related.^17^

As Wales records only gross secondary care expenditure as a function of the underlying diagnosis group of circulatory diseases, NHS England's unit costs for A&E activity and outpatient activity measures were applied to Welsh CV-related activity levels for A&E and outpatient care respectively, and these products were then subtracted from the total Welsh CVD-related secondary care amount.

#### Outpatient specialist care

Outpatient specialist care was captured by taking the product of outpatient activity and per-consultation cost for specialties that are related to CVD. The list of specialties included in this tally is outlined in online Appendix B, alongside their activity levels and unit costs. Generalist specialties that do see CVD in their practice, but where CVD is not dominant were adjusted by a proportion as measured in a pan-European survey of general internists regarding their practice case-mix, at 17.9%.^17^ Stroke and transient ischaemic attack (TIA) services were not separately available from Neurology services in the Scottish and Northern Irish activity levels. The NHS England ratio of Stroke and TIA services to Neurology services (30%) was applied to Scottish and Northern Irish Neurology activities to estimate the neurovascular outpatient burden in these countries. Vascular surgery was not separated from general surgery in Northern Ireland. The ratio of vascular surgery to general surgery in NHS England (7.2%) was applied to Northern Irish General Surgery consultations to estimate the outpatient burden of peripheral vascular disease.

Activity levels and unit costs were only available for England, Scotland, and Northern Ireland. The ratio of outpatient costs attributable to CVD to total NHS England secondary care costs (Inpatient, Outpatient, and A&E care) was applied to the Welsh Secondary Care budget attributable to Circulatory Disease as an estimate of Welsh CVD outpatient costs.

#### Accident & emergency care

The number of A&E visits that were attributable to CVD were estimated for England through the extraction of Systematized Nomenclature of Medicine Clinical Terms codes recorded for the country. The codes that were used can be found in online Appendix C. Consultations that were not coded or had invalid codes had the proportion of CVD to all coded consultations applied to estimate the CVD-related burden of these codes. The proportion of total A&E visits attributable to CVD for NHS England was then applied to A&E activity for NHS Scotland and NHS Northern Ireland. Weighted unit costs from NHS England were then applied to the English, Scottish, and Northern Irish A&E activities. Weighted unit costs can be found in *Table 2*. For Wales, the A&E proportion of total secondary costs in NHS England was applied to the Welsh Secondary Care budget attributable to circulatory conditions.

**Table 2:** Summary of total direct costs of cardiovascular diseases in the UK

	2015 EHN estimate (£)	2019/20 Estimate (£)	2020/21 Estimate (£)	2021/22 Estimate (£)
**Inpatient care (UK)**	**5 449 545 832.19**	**5 902 562 012.59**	**6 258 547 282.76**	**6 732 456 821.82**
England		4 829 042 918.01	5 252 498 249.60	5 575 044 033.98
Scotland		555 593 708.52	484 738 462.74	583 225 030.98
Wales^a^		381 061 330.72	408 885 361.77	430 622 677.32
Northern Ireland		136 864 055.34	112 425 208.64	143 565 079.54
**Outpatient care (UK)**	**899 427 786.19**	**900 995 134.97**	**924 269 717.97**	**1 010 695 676.09**
England		772 226 533.94	811 142 628.75	883 921 864.36
Scotland		44 061 589.33	38 591 873.64	43 584 349.13
Wales^a^		59 363 061.84	61 577 941.01	66 795 502.00
Northern Ireland		25 343 949.86	12 957 274.58	16 393 960.59
**Accident & emergency care (UK)**	**333 955 900.58**	**357 042 291.74**	**392 362 590.87**	**327 626 962.37**
England		308 593 003.49	339 379 213.24	286 976 900.56
Scotland		23 118 333.15	24 470 618.16	18 713 154.99
Wales^a^		13 881 834.01	12 890 470.59	9 583 101.19
Northern Ireland		11 449 121.09	15 622 288.87	12 353 805.64
**Primary care (UK)**	**1 372 554 805.84**	**1 423 677 763.98**	**1 265 861 038.04**	**1 555 641 199.16**
England		1 126 296 422.13	976 596 690.54	1 260 899 714.88
Scotland^a^		129 700 817.24	118 485 741.33	111 904 484.28
Wales^a^		131 359 558.08	132 930 648.90	141 713 000.00
Northern Ireland^a^		36 320 966.53	37 847 957.27	41 124 000.00
**Medications (UK)**	**2 288 700 449.83**	**1 805 967 156.62**	**1 853 979 414.91**	**1 940 427 596.98**
England		1 497 733 264.99	1 546 702 608.69	1 615 340 915.00
Scotland		153 479 164.53	148 020 946.28	158 996 452.13
Wales		87 273 612.44	92 908 334.05	98 434 848.00
Northern Ireland		67 481 114.65	66 347 525.89	67 655 381.85
**Total direct costs without LTC/devices**	**10 344 184 774.63**	**10 390 244 359.89**	**10 695 020 044.55**	**11 566 848 256.42**
**Medical devices (UK)**		**307 116 365.46**	**243 893 010.21**	**404 371 349.20**
England		258 794 328.22	205 518 607.38	340 747 102.52
Scotland^b^		25 118 962.70	19 947 941.94	33 073 420.95
Wales^b^		14 496 289.33	11 512 065.26	19 086 850.24
Northern Ireland^b^		8 706 785.21	6 914 395.63	11 463 975.48
**Long-term care (UK)**		**3 903 440 803.45**	**4 384 334 226.00**	**4 648 757 713.68**
England^c^		3 296 217 807.93	3 700 433 411.65	3 919 968 549.09
Stroke^c^		1 614 539 289.95	1 894 186 114.56	2 023 408 887.14
Vascular dementia^c^		1 681 678 517.98	1 806 247 297.09	1 896 559 661.95
Scotland^c^		333 532 308.24	371 773 342.28	395 575 225.37
Stroke^c^		196 506 651.22	228 519 246.30	245 158 424.60
Vascular dementia^c^		137 025 657.02	143 254 095.98	150 416 800.78
Wales^c^		189 901 379.40	214 977 770.95	227 518 600.46
Stroke^c^		93 983 419.49	111 585 684.29	118 956 909.46
Vascular dementia^c^		95 917 959.91	103 392 086.66	108 561 690.99
Northern Ireland^c^		83 789 307.88	97 149 701.12	105 695 338.75
Stroke^c^		41 435 922.98	52 304 940.64	58 608 340.25
Vascular dementia^c^		42 353 384.90	44 844 760.48	47 086 998.50
**Total direct costs with LTC/devices**		**14 600 801 528.80**	**15 323 247 280.76**	**16 619 977 319.30**

All costs are represented in real terms in GBP in 2022. Medical devices and long-term care costs were not captured in 2015, and have been included without 2015 comparators.

EHN, European Heart Network; Inpatient care, hospital-based care; Outpatient care, clinic-based specialist care; Accident and Emergency, emergency department attendances; Primary care, clinic-based general practitioner or family physician care; Medications, all cardiovascular prescription medications; Medical devices, deployed cardiovascular medical devices e.g. pacemakers, stents; Long-term care, institutionalized care settings e.g. nursing homes.

a Only aggregate costs were available.

b Projected estimates based on nation's population compared with England.

c Modelled cost.

#### Primary care costs

Primary care activity and spending was tracked across all four nations, however there were no publicly available databases that allowed for an estimate of the proportion of consultations that were attributable to CVD. The proportion of consultations attributable to hypertension as captured in a previous study (12% in 2017) was used as a proxy.^18^ This figure was used as hypertension was the most common CVD reason for primary care in a previous study used to estimate the proportion of primary care consultations due to CVD.^19^ Unit costs for primary care consultations were taken from the Personal Social Services Research Unit's *Unit Costs of Health and Social Care* for each respective year analysed.^20^ Unit costs were only applied for NHS England, as all other nations reported aggregate primary care spending, to which the estimated proportion attributable to CVD was applied.

#### Long-term residential and nursing home care

LTC costs were modelled for post-stroke care and VaD, as the top diagnoses leading to institutionalization were dementia and stroke.^6^ There were no publicly available data capturing the admitting diagnosis to LTC overall in the UK. Thus, for post-stroke care, the Sentinel Stroke National Audit Programme (SSNAP) database was used to derive transition probabilities for patients after stroke and a Markov model was generated to determine the estimated number of individuals receiving LTC in 2019.^21^ The Markov Model is shown in online Appendix D1 and the Transition Probabilities used are shown in online Appendix D2. The cost of VaD was estimated by applying the proportion of all dementia that was thought to be due to VaD (17%) to the total social care costs that were modelled in the 2019 Alzheimer's UK report on the economic burden of dementia in the UK.^22^

#### Medication costs

Medication costs were directly extracted from each of the four countries using the British National Formulary Cardiovascular Chapter (Chapter 02).^23^ The total costs for ingredients and dispensing are captured within the Prescription Cost Analysis document for each country.

#### Medical device costs

High-cost medical devices are not captured within the HRG codes that represent their use or implantation, such as endovascular grafts for aortic repair. These devices are tracked separately within the NHS England National Cost Collection document and tabulated. Total expenditures for Northern Ireland, Scotland, and Wales were determined by adjusting the NHS England medical device expenditure for each nation's population per the Office of National Statistics (ONS) Census. The included devices can be seen in online Appendix E.

### Indirect costs

#### Costs and productivity losses to morbidity

Morbidity-related costs and losses were captured through a combination of sickness days due to circulatory disease as captured by the ONS, the employment service allowance (ESA), and the disability living allowance (DLA).^24–27^ The number of total sickness days recorded was multiplied by the average daily UK wage for each respective year. The average weekly payment for ESA and DLA were multiplied by the number of claimants per quarter, and adjusted for the amount of time the claim had already been in place. For ESA, the CVDs category was used to identify CVD-related claims. For DLA, claims related to Heart Disease, Cerebrovascular Disease, Peripheral Vascular Disease, and Dementia (adjusted for the prevalence of VaD) were used.

#### Production losses to mortality

Production losses to mortality were captured assuming a working age between 15 and 65 years old. The ONS captures all deaths secondary to CVD, organized by age of death and gender. Age and gender-specific employment rates and average wages were then applied to these deaths and serially discounted by the recommended 3.5% per annum as per His Majesty's Treasury, up to the retirement age of 65.^28^ The age- and gender-specific employment rates and wages applied to cardiovascular deaths can be seen in online Appendix F.

#### Informal care

Informal care costs were estimated using the results from the Survey of Health, Aging, and Retirement in Europe (SHARE) using the same methodology as the EHN’s COI study in CVD performed in 2017.^5,29^ Unfortunately, similar survey data in the UK was unavailable. SHARE captures information from individuals over 50 years of age regarding their health status, presence of chronic diseases, as well as the frequency and duration of help that respondents received from those within and outside of their household. SHARE also recorded who helped the individual. SHARE, as it was based in continental Europe, does not contain direct information with respect to the UK. Rather, the responses from Austria, Belgium, France, Germany, Ireland, Luxembourg, and the Netherlands have been combined to represent the UK, as done in EHN's Cardiovascular Statistics study.^5^

The estimated economic burden of informal care was estimated to be the product of each of the following components:

1. The population within each nation that was greater than 50 years old.
2. The SHARE prevalence of heart attack, hypertension, and stroke (the available CVD-related diagnoses within the survey).
3. The marginal effect of having a CVD-related diagnosis as derived from a logistic regression on the development of a severe disability, controlling for age, gender, and the number of comorbidities the respondent had.
4. The marginal effect of having a CVD-related diagnosis derived from a logistic regression on requiring help from in-household or out-of-household help with tasks, controlling for age, gender, number of comorbidities, and household size.
5. The marginal effect of CVD derived from an ordered logistic regression on requiring 1, 2, or 3 carers, and how often (daily, weekly, monthly, annually) these individuals required help, controlling for age, gender, number of comorbidities, and household size.
6. The total number of hours of care provided to each individual by carers 1, 2, and 3, respectively.
7. The projected hourly wage of the individual providing care:
  - For unemployed individuals (for those respondents who were >65 years old, it was assumed that siblings, spouses, friends, and parents would similarly be over the retirement age), the national minimum wage was used for each respective year.
  - Employed individuals—for all other individuals, providing care, the average hourly wage for all employed individuals in the UK was used for each respective year.

### Discounting and currency conversion

To make results comparable to previously done COI studies, currencies were converted to Great British Pound Sterling (GBP) at the mid-year floating rate for the fiscal year of publication as available from the European Central Bank.^30^ Discounting of future costs was done as per His Majesty's Treasury (HM Treasury) recommendation of 3.5% per annum.^37^ Adjustment for inflation was done according to the gross domestic product deflator from HM Treasury.^31^ All figures and data are presented in real terms in 2022.

### One-way sensitivity analysis

One-way sensitivity analysis was performed to determine the impact of uncertainty of individual variables on the total COI estimate (including medical devices and LTC in the total). The variables tested, their base-case, lower, and upper-limit values, as well as their reference sources are included in online Appendix G.

### Probabilistic sensitivity analysis

Probabilistic sensitivity analysis (PSA) was performed for the informal care cost, stroke LTC cost, and productivity loss from mortality models. A Monte Carlo simulation was run, with a total of 1000 simulated trials for each respective model. The list of variables and their respective distributions used in the analysis are shown in online Appendix H for informal care costs, online Appendix D2 for stroke LTC COSTS, and online Appendix F for the mortality costs.

## Results

### Cost-of-illness

#### Direct costs

The total direct costs are illustrated, by nation, in *Table 2*. For 2021/22, inpatient services comprise the largest component of costs in the UK at £6.732 bn. Long-term residential care was the next greatest cost at £4.649 bn. Medications comprised £1.940 bn. Primary care and outpatient specialist care follow at £1.556 bn and £1.011 bn, respectively. A&E and medical devices comprise the remainder at £327.6 mn and £404.4 mn, respectively. A breakdown of costs by nation is also shown in *Table 2*, alongside a comparison with the estimate from the 2017 EHN COI study.^5^ All units of measurement, the number of units consumed, and average unit costs are outlined in *Table 2*. Overall, there is an estimated increase between 2019/20 and 2021/22 from £10.390 bn to £11.567 bn for direct costs, excluding medical devices and LTC costs. Including these new costs, the direct costs increased from 14.601 bn in 2019/20 to £16.620 bn for 2021/22.

#### Indirect costs

The total indirect costs are illustrated, by nation, in *Table 3*. All units of measurement, units consumed, and average unit costs are included in this table. Losses from morbidity in the UK amounted to £1.481 bn in 2021/22, losses from mortality amounted to £4.544 bn, and informal care costs totalled £6.377 bn. This marks an increase between 2019/20 and 2021/22 for total indirect costs from £11.690 bn to £12.402 bn in 2022 real terms.

**Table 3:** Summary of indirect costs, by Nation

	2015 EHN estimate (£)	2019/20 Estimate (£)	2020/21 Estimate (£)	2021/22 Estimate (£)
**Losses from morbidity (UK)**	**2 134 749 896.72**	**1 804 975 954.90**	**1 371 129 207.87**	**1 480 608 481.76**
England		1 419 679 848.96	1 059 000 522.69	1 175 542 772.43
Scotland		192 876 488.60	149 031 457.97	136 032 383.51
Wales		114 661 775.69	98 244 014.10	101 492 987.92
Northern Ireland		77 757 841.65	64 853 213.10	67 540 337.90
**Losses from mortality (UK)**	**5 196 227 187.18**	**4 170 139 187.51**	**4 472 253 912.71**	**4 543 976 274.97**
England		3 363 716 186.66	3 622 785 839.23	3 669 492 556.71
Scotland		456 442 067.99	485 383 301.09	504 266 240.34
Wales		234 007 757.42	247 939 694.93	257 445 031.12
Northern Ireland		115 973 175.44	116 145 077.46	112 772 446.81
**Informal care costs (UK)**	**4 663 386 830.99**	**5 714 817 518.95**	**5 433 376 101.67**	**6 376 929 218.16**
England		4 772 804 883.59	4 539 578 016.63	5 326 626 027.01
Scotland		495 126 560.14	469 157 142.94	552 098 576.72
Wales		293 301 540.45	278 197 853.56	323 745 534.30
Northern Ireland		153 584 534.77	146 443 088.54	174 459 080.12
**Total indirect costs**	**11 994 363 914.90**	**11 689 932 661.36**	**11 276 759 222.25**	**12 401 513 974.89**

All costs are represented in real terms in GBP in 2022. Losses from morbidity are a combination of disability payments and time taken off of work. Losses from mortality are the total lost working potential of the individual up to the retirement age of 65, adjusted for employment status, gender, and age. Informal care costs are the costs associated with the unpaid caretakers of individuals, adjusted for age and employment status.

EHN, European Heart Network; ESA, employment support allowance; DLA, disability living allowance; CVD, cardiovascular disease.

### Informal care model estimation

The marginal effects of CVD on the development of a severe disability, requiring help from an in-house or out-of-house individual, the number of carers that the individual requires, and how frequently these carers are in contact with the survey respondent are summarized in *Table 4*. The marginal effect of CVD on becoming severely disabled was 0.1448 (95% CI 0.1375–0.1522), receiving in-house help was 0.1124 (95% CI 0.1041–0.1206), and on receiving out-of-house help was 0.2427 (95% CI 0.2338–0.2516). The marginal effect of CVD on requiring a first carer who would be in contact daily was 0.1233 (95% CI 0.1144–0.1322), in contact weekly was 0.2812 (95% CI 0.2691–0.2933), in contact monthly was 0.2081 (95% CI 0.1986–0.2176), and annually was 0.3874 (95% CI 0.3715–0.4033). The marginal effect of CVD on requiring a second carer who would be in contact daily was 0.0892 (95% CI 0.0780–0.1003), in contact weekly was 0.3107 (95% CI 0.2910–0.3303), in contact monthly was 0.2525 (95% CI 0.2369–0.2681), and annually was 0.3477 (95% CI 0.3248–0.3707). The marginal effect of CVD on requiring a third carer who would be in contact daily was 0.0818 (95% CI 0.3248–0.3707), in contact weekly was 0.3016 (95% CI 0.2707–0.3325), in contact monthly was 0.2808 (95% CI 0.2546–0.3070), and annually was 0.3358 (95% CI 0.3004–0.3713).

**Table 4:** Summary of marginal effects of CVD on key variables for modelling informal care costs

Effect	Margin	SE	*P-value*	95% CI	Pseudo-*R*^2^
Severe disability	0.1448	0.003754	<0.0005	0.1375–0.1522	0.1033
Receiving in-household help	0.1124	0.004209	<0.0005	0.1041–0.1206	0.0356
Receiving out-of-household help	0.2427	0.004543	<0.0005	0.2338–0.2516	0.0318
Requiring first carer					0.0382
Daily	0.1233	0.004540	<0.0005	0.1144–0.1322	
Weekly	0.2812	0.006164	<0.0005	0.2691–0.2933	
Monthly	0.2081	0.004866	<0.0005	0.1986–0.2176	
Annually	0.3874	0.008089	<0.0005	0.3715–0.4033	
Requiring second carer					
Daily	0.0892	0.005699	<0.0005	0.0780–0.1003	0.0282
Weekly	0.3107	0.010005	<0.0005	0.2910–0.3303	
Monthly	0.2525	0.007954	<0.0005	0.2369–0.2681	
Annually	0.3477	0.011712	<0.0005	0.3248–0.3707	
Requiring third carer					0.0312
Daily	0.0818	0.008625	<0.0005	0.0649–0.0987	
Weekly	0.3016	0.015777	<0.0005	0.2707–0.3325	
Monthly	0.2808	0.013386	<0.0005	0.2546–0.3070	
Annually	0.3358	0.018088	<0.0005	0.3004–0.3713	

SE, standard error; CI, confidence interval.

### Sensitivity analyses

#### One-way sensitivity analysis

**Figure 1:**
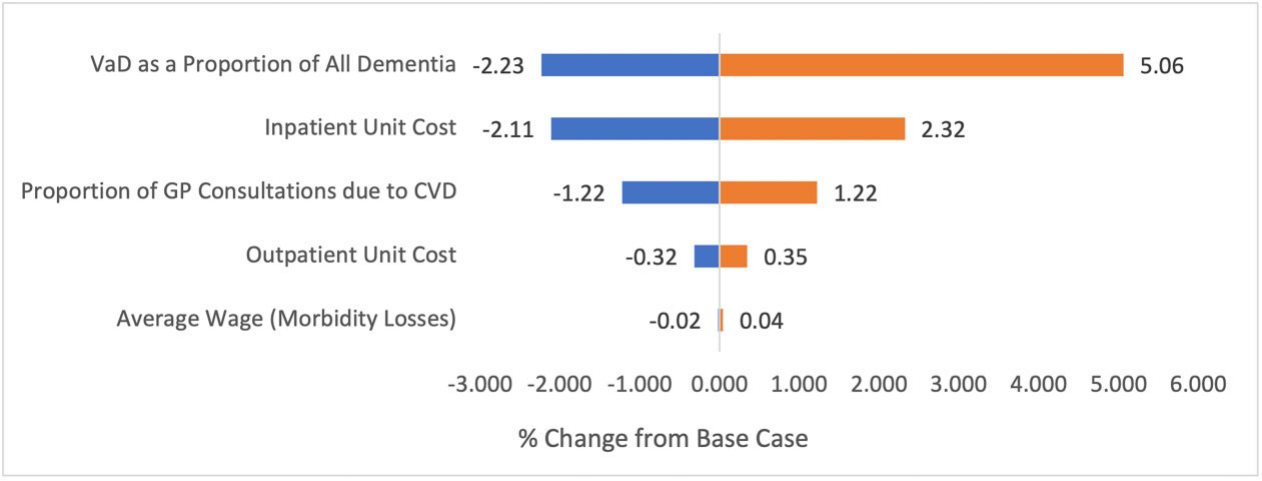
One-way sensitivity analysis of deterministic models. Figures are expressed as % change from the base-case total-UK cost-of-illness of cardiovascular disease in 2021/22, including long-term care and medical device costs.

The Tornado diagram illustrating the one-way sensitivity analysis of deterministic costs is shown in *Figure 1*. The values and references for this analysis can be found in online Appendix G. The greatest impact was the proportion of all dementia attributable to VaD, with a percentage change of −2.23% to +5.06%, followed by the unit cost of inpatient care, with a change of −2.11% to +2.32%. Changing proportion of GP consultations attributable to CVD altered the final estimate by −1.22% to +1.22%. Outpatient unit costs and the uncertainty around average wage had a smaller effect with −0.32% to +0.35% and −0.02% to +0.04%, respectively.

#### Stroke LTC model probabilistic sensitivity analysis

**Figure 2:**
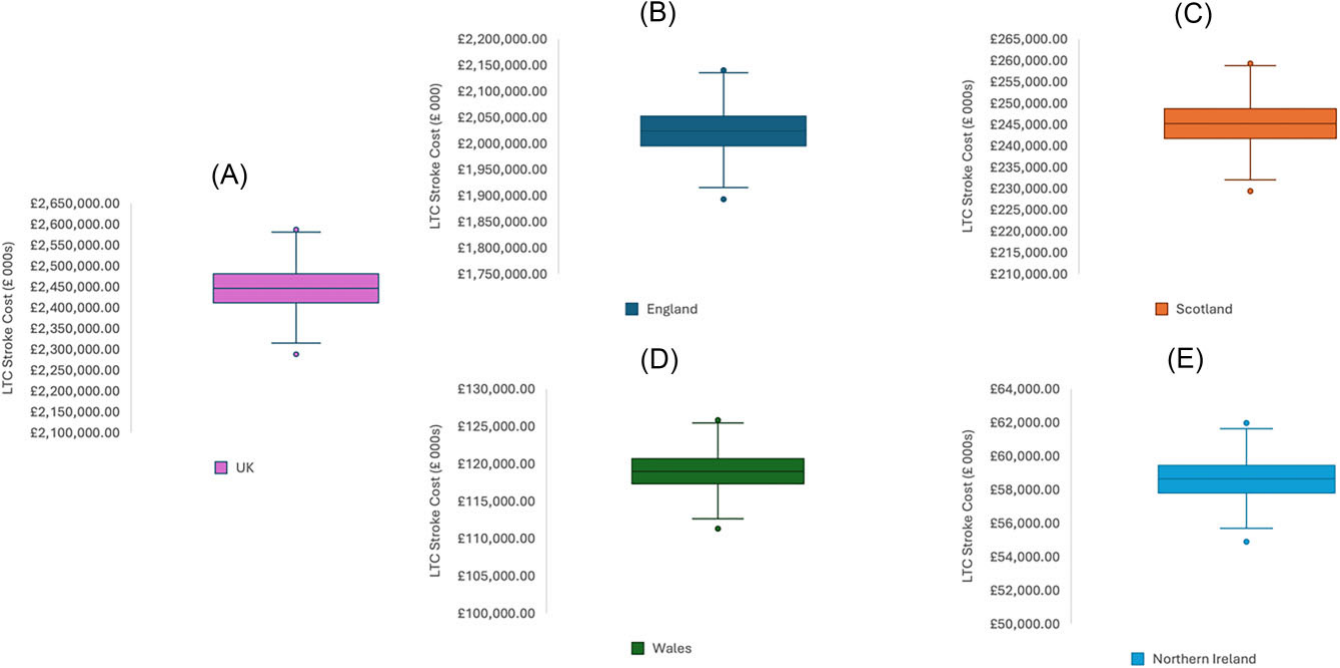
Distribution of PSA trial results for long-term care stroke care costs for the UK (A), England (B), Scotland (C), Wales (D), and Northern Ireland (E). Box-plot whiskers represent the minimum and maximum values. The lower bound of the box itself is the 25th percentile. The upper limit of the box is the 75th percentile. The intersecting bar is representative of the median value.

The distributions of modelled costs for the Stroke LTC Model for 2021/22 are shown in *Figure 2*. For the UK (*Figure 2A*), the median value was £2.446 bn (IQR £2.412 bn–£2.481 bn). For England (*Figure 2B*), the median value was £2.023 bn (IQR £1.995 bn–£2.052 bn). For Scotland (*Figure 2C*), the median value was £245.1 mn (IQR £241.7 mn–£248.7 mn). For Wales (*Figure 2D*), the median value was £119.0 mn (IQR £117.3 mn–£120.6 mn). For Northern Ireland, the median was £58.6 mn (IQR £57.8 mn–£59.4 mn).

#### Informal care model probabilistic sensitivity analysis

**Figure 3:**
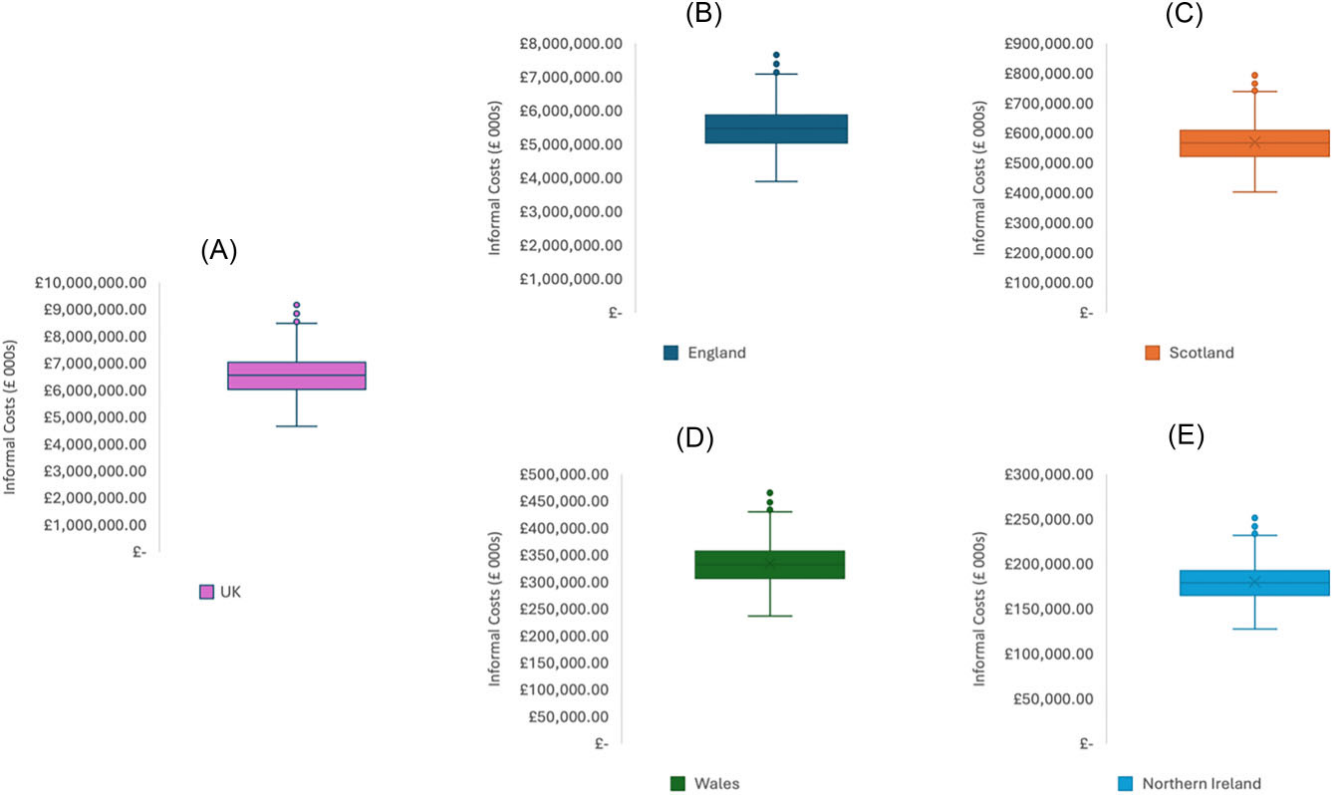
Distribution of probabilistic sensitivity analysis trial results for Informal Care Costs for the UK (A), England (B), Scotland (C), Wales (D), and Northern Ireland (E). Box-plot whiskers represent the minimum and maximum values. The lower bound of the box itself is the 25th percentile. The upper limit of the box is the 75th percentile. The intersecting bar is representative of the median value.

The distributions of modelled costs for informal care for 2021/22 are shown in *Figure 3*, by nation. For the UK (*Figure 3A*), the median value was £6.550 bn (IQR £6.037 bn–£7.033 bn). For England (*Figure 3B*), the median value was £5.472 bn (IQR £5.042 bn–£5.875 bn). For Scotland (*Figure 3C*), the median value was £566.8 mn (IQR £522.7 mn–£609.1 mn). For Wales (*Figure 3D*), the median value was £332.4 mn (IQR £306.7 mn–£357.1 mn). For Northern Ireland (*Figure 3E*), the median was £179.2 mn (IQR £165.3 mn–£192.6 mn).

#### Mortality model probabilistic sensitivity analysis

**Figure 4:**
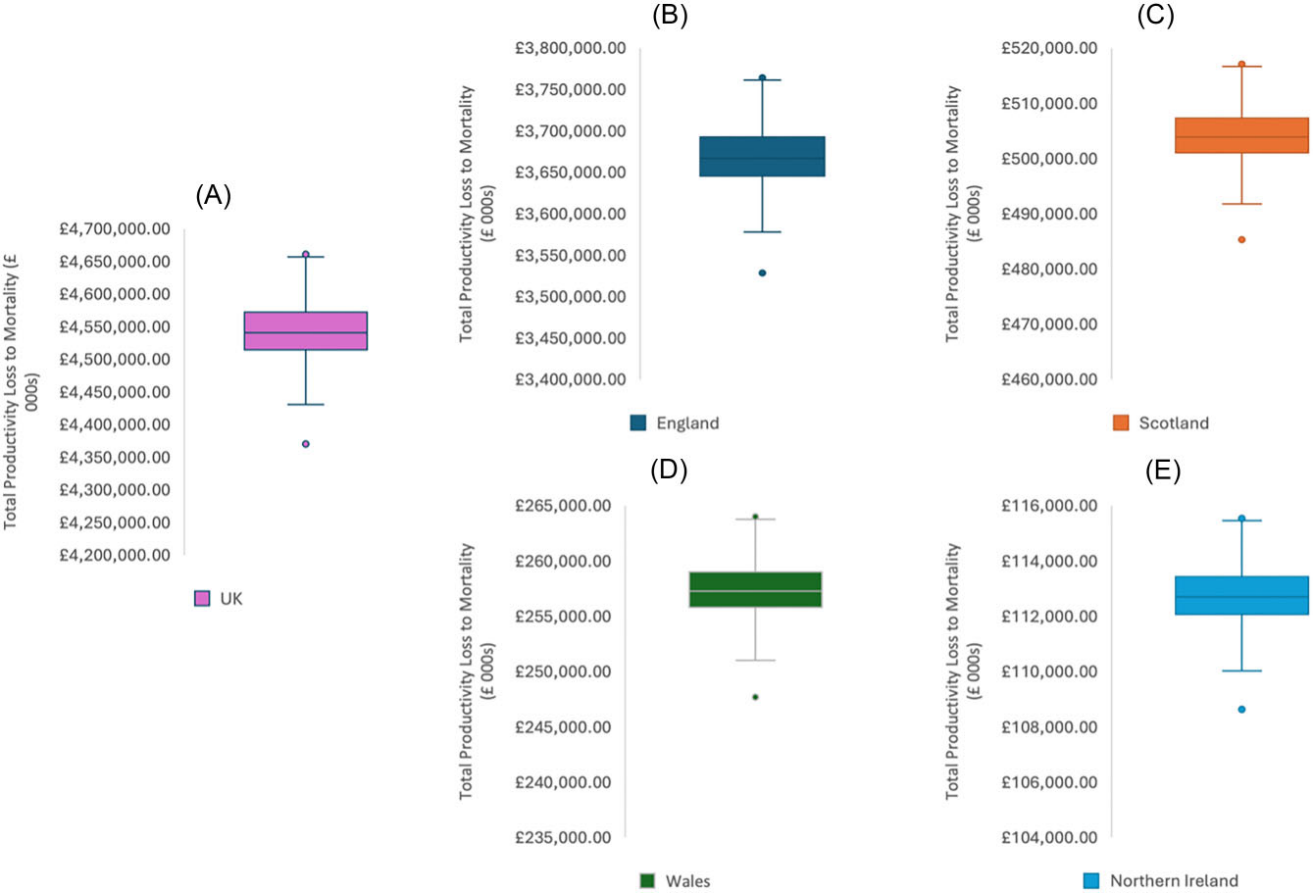
Distribution of probabilistic sensitivity analysis trial results for Productivity Loss to Mortality for the UK (A), England (B), Scotland (C), Wales (D), and Northern Ireland (E). Box-plot whiskers represent the minimum and maximum values. The lower bound of the box itself is the 25th percentile. The upper limit of the box is the 75th percentile. The intersecting bar is representative of the median value.

The distributions of costs for the mortality model for 2021/22 are shown in *Figure 4*, by nation. For the UK (*Figure 4A*), the median value was £4.540 bn (IQR £4.514 bn–£4.572 bn). For England (*Figure 4B*), the median value was £3.667 bn (IQR £3.645 bn–£3.667 bn). For Scotland (*Figure 4C*), the median value was £503.9 mn (IQR £501.0 mn–£507.3 mn). For Wales (*Figure 4D*), the median value was £257.2 mn (IQR £255.8 mn–£259.0). For Northern Ireland (*Figure 4E*), the median was £112.7 mn (IQR £112.1 mn–£113.4 mn).

#### Impact of model uncertainty on cost-of-illness estimate

**Figure 5:**
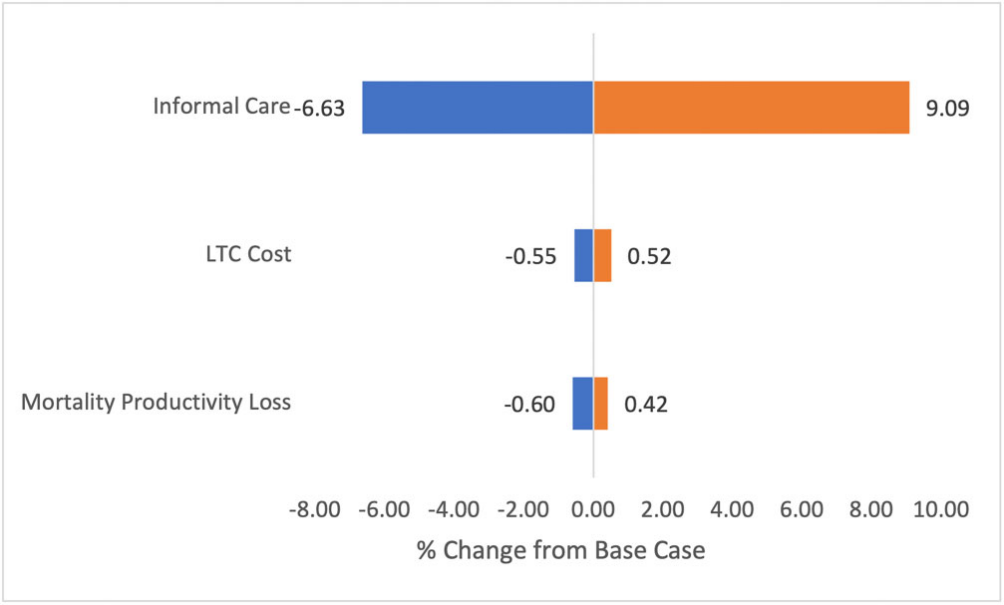
Impact of model uncertainty in informal care costs, long-term care stroke cost, and productivity loss to mortality on overall cost-of-illness of CVD in 2021/22. Figure is presented as change from the base case including LTC and medical devices, in %.

To determine the overall impact of the uncertainty of the LTC stroke, informal care, and mortality models on the final COI estimate, the minimum and maximum UK PSA values were applied to generate the Tornado diagram in *Figure 5*. Here, we see the impact of uncertainty within the informal care model is greatest, varying the final COI estimate from −6.63% to +9.09%. The uncertainty within the LTC cost and mortality productivity loss models is much smaller, from −0.55% to +0.52%, and −0.60% to +0.42%, respectively.

## Discussion

### Cost of illness results

The results indicate that the overall cost of CVD in the UK in 2021/22 is £29.021 bn, which has partly been driven by the cost of inpatient care, which increased by 23.5% (£1.28 bn) over since 2015. This is somewhat offset by a decrease in medication costs of 15.3% (£348.3 mn) over the same period. The addition of medical devices and LTC costs related to stroke and VaD have added a total of £5.053 bn—a significant figure, amounting to 40% of the other direct medical costs of CVD. Through the COVID-19 years, costs increase significantly, though with different trajectories for each subtype of care provided. We see steady increases in inpatient care, outpatient care, and medication costs. Meanwhile, A&E care peaks during the first year of the COVID-19 pandemic in 2020/2021 and primary care costs stagnant over that same period.

Informal costs increased from 2015, with a rise of £1.714 bn over 7 years. This figure, however, may not be reliable given the differences in modelling taken. In particular, the greatest change from the 2015 EHN estimate is in informal care costs, which have increased by more than 36%. The sensitivity analysis, however, illustrates that uncertainty in the informal care model may account for a 19% variation in the base-case COI including LTC and devices. A recent paper by Luengo *et al.* 2024 has estimated the productivity losses from premature cardiovascular mortality in the UK at €7.25 bn in 2018, equivalent to £6.10 bn in 2022 real terms after adjusting for exchange and inflation.^30–32^ This is higher than our estimate of £4.54 bn in 2021/2022, however the model used in Luengo *et al.* 2024 assumes some individuals work until the age of 79 years old rather than the standard retirement age most commonly used in Europe of 65 years old. Moreover, the effect of the COVID-19 pandemic on service delivery makes it difficult to compare these two time periods. Overall, the impact of premature mortality from CVD on productivity losses is significant in both analyses, and worthy of mitigation through increased investment in prevention and treatment strategies.

Overall, CVD-related direct costs, as estimated in this study (£16.620 bn), comprised 7.3% of total public UK healthcare spending (£228.289 bn) in 2021/22.^33^ The estimated LTC cost of CVD (£4.649 bn), when isolated from other direct care costs, comprised 10.7% of total UK LTC spending in 2021(22).^8^ Although this difference in proportion of spending may reflect the natural history of vascular disease and the significant morbidity associated with it, the significantly larger burden that CVD has on the LTC as compared with the healthcare as a whole suggests that more can be done in the areas of prevention, early diagnosis, and treatment.

### Model uncertainty

The sensitivity analysis that was performed indicates that the estimate is most sensitive to the modelling of informal care, with a 15.7% variance in the final cost estimate for informal care. The proportion of all dementia as VaD, the proportion of all GP visits as CVD-related visits, and inpatient unit costs are also significant drivers of uncertainty. Should we take the extreme values of these sensitivity analyses, it would amount to a total change in the final estimate by −£3.967 bn to +£5.514 bn. Much of this uncertainty is secondary to the limitations of the models used to estimate these values, which will be discussed in the upcoming section.

### Model limitations

There are several limitations to the models applied to estimate COI in this study. Several assumptions were made in the cost-capturing methodology, which should be acknowledged. The major limitations in the methodology fall in the cost estimates for primary care, LTC, and informal care cost categories. Each will be discussed in turn.

Although primary care visits are captured across the UK, it is difficult to capture what proportion of these consultations and General Practitioner (GP) time can be attributable to CVD. This is for several reasons. First, patients often go to the GP with multiple complaints, particularly for those who are multi-morbid.^34^ Second, GP practices are typically privately operated, and there is no direct reporting to the NHS regarding the primary complaint of the patient when consulting their GP. Instead of this public reporting, some private databases have been created to track more detailed primary care information from participating practices. Data from these initiatives are highly curated but do require significant financial resources to access. Instead, this study has used an estimation of the number of GP consultations in the UK that are attributable to hypertension to represent total CVD burden in primary care. This may be an underestimation, however the last published estimate of total CVD burden on primary care, which has been used in both the 2017 Wilkins and the 2006 Luengo-Fernandez study, was done using 1991–92 survey data of GP practices. This places the total burden of CVD on primary care consultations at 9%, already lower than the 12% of all consultations estimated for hypertension.^4,5,18,19^ Thus, this 12% figure has been used, acknowledging that it may be an underestimate of CVD burden on GP practices. However, this 12% figure is similar to other estimates used for the proportion of primary care appointments that are CVD-related, which varied from 7.8% in Spain to 14.4% in Estonia in a recent CVD COI study focused on the European Union (EU).^35^ Ideally in future studies, more granular primary care data would allow for a more representative estimate.

To capture LTC in this study, the two major cost components identified were stroke and VaD. However, primary cardiac diseases, particularly heart failure (HF), also contribute a significant portion to overall CVD burden, though their contribution to LTC utilization in the UK is less clear. A previous study of the COI of HF in the UK used the proportion of all hospital admissions attributable to HF and applied this to the total number of patients discharged to a LTC facility as an estimate of the number of new nursing-home admissions secondary to HF.^36^ This figure was estimated at £105.7 mn in 1995. Unfortunately, recent data documenting the discharge disposition of admitted HF patients were not available. A study in the Netherlands estimated that the prevalence of HF in nursing homes was 33%, though it is not clear for which proportion of these patients the primary causative diagnosis for admission to LTC was HF.^37^ This paucity of data and the multi-system nature of HF, often involving the cardiovascular, respiratory, and renal systems also would make modelling of these costs highly uncertain.^38^ Thus, in this study, the contribution of HF to LTC burden was omitted, thus generating a conservative estimate of overall CVD COI.

In the LTC stroke modelling, there is a limitation in that the transition probabilities for stroke care are not stable over time. With improving technologies and processes applied throughout the stroke care pathway, mortality and care-home admissions have been declining annually, with increasing numbers of patients being discharged home either directly or through Early Stroke Discharge and Community Resource Teams.^21^ The applicability of the transition probabilities applied within this model is limited to the timeframe in which the model is applied and will not be accurate in future COI studies as the dynamic field of stroke care continues to innovate.

Within the LTC VaD estimate, this study applied the proportion of dementia attributable to VaD to a modelled economic burden estimate from another study.^22^ This application assumes that VaD patients consume resources at a similar rate and intensity as compared with their Alzheimer's disease (AD) and other dementia counterparts. This, however, is not necessarily the case. VaD patients typically have multiple vascular comorbidities such as coronary artery disease and stroke, these patients tend to consume more healthcare resources in the short-term and tend to have a lower life expectancy as compared to those with AD.^39,40^ There is a range of estimated differences in annual medical care costs, with VaD being 36–70% more costly on an annual basis as compared with AD, though another recent meta-analysis estimates that VaD patients have a 45% shorter survival from diagnosis as compared to their AD counterparts.^39–41^ Thus, the estimate of economic burden of VaD in LTC may be conservative, however the increase in annual cost of VaD patients may be offset by lower survival. Our decision to only model LTC costs of VaD may also produce conservative estimates of the economic burden of CVD as increased CVD prevalence is associated with increased risk of non-VaD.^14,42^ However, non-VaDs have many non-CVD-related risk factors, and it was not possible to estimate the specific contribution of CVD to the development and progression of these diseases.^43^

The estimation of informal care costs in this study was completely dependent on survey data collected outside of the UK. Although previous studies have taken this approach, native-UK data would be preferable. In addition to this, recall bias and the fact that many of the individuals in the survey cohort required a family member or delegate to answer the questions for them may also have limited data quality. Should future UK-based data be available for later COI studies, these should be used as these would better reflect the realities of life for CVD patients in the UK. All methods of informal care estimation require caregiver input and an estimation of disease prevalence in the population studied. Dedicated survey data capturing both elements simultaneously would provide the most accurate estimation of informal care costs. Our use of European survey data on informal care therefore raises potential concerns of generalizability of the data to the UK context.

More generally, in many cases the data from England were more detailed than those in the other UK constituent countries. This meant we had to extrapolate England's estimates to Northern Ireland, Scotland, and Wales for several model inputs, including medical device spending, A&E attendances, and primary care costs. While this is an imperfect solution, the lack of comparative data across UK constituent countries necessitates it. However, this approach does raise concerns regarding regional accuracy, particularly due to demographic and socioeconomic differences between populations in England, Scotland, Wales, and Northern Ireland.^44^

Finally, the period analysed in this study included the peak years of the COVID-19 pandemic. The data for these years were the most recently available, as average unit costs from NHS England are not currently available beyond the 2021/22 fiscal year. While there were significant changes to the pattern of healthcare utilisation in the UK during our period of analysis with a significant decline in healthcare services utilized near the beginning of the COVID-19 pandemic, most services had returned to pre-pandemic utilization levels by 2021/22.^45–47^ Given the significant changes to utilisation patterns through the time period included in this study, trends must be interpreted with caution as the health system recovers from this significant shock. When more recent data are available for all four nations, re-analysis would be helpful to determine underlying trends in the COI of CVD in a pandemic-recovery setting.

## Conclusion

To conclude, there is a significant economic burden of CVD in the UK, with noted growth in its COI particularly in indirect costs such as production losses and informal care. Healthcare expenditures have increased over a 7-year period in real terms, with the caveat that estimation techniques varied slightly between previous studies and this paper. The addition of LTC and medical devices to the existing core components of previous CVD-related COI studies performed in the UK setting has added £4.5 bn to the societal cost of CVD in 2021/22 and should be considered an important costing component in future COIs performed in this disease group. The burden of disease on public expenditure budgets is significantly higher in the LTC space as compared with the overall healthcare space—the interpretation of this malalignment is up for debate, but could indicate further opportunities for investment in prevention, early diagnosis, and definitive management. Although COI studies are unable to provide direct guidance to policymakers regarding the allocation of healthcare resources, they do provide an assessment of the magnitude of disease impact. With serial assessments, they may also be helpful in interpreting how policy interventions within the space have impacted spending. In future, concurrent COI studies in different disease groups, employing the same estimation methodology would be helpful as one piece of evidence to aid decision-makers in policymaking.

## Supplementary Material

qcaf011_Supplemental_File
