# Supplementary material for: Economic burden of cardiovascular disease in the United Kingdom
Source: Eur Heart J Qual Care Clin Outcomes. 2025 Feb 24;11(5):678–90. doi: 10.1093/ehjqcco/qcaf011 (PMC12342905; doi:10.1093/ehjqcco/qcaf011)
Supplement: qcaf011_Supplemental_File [file qcaf011_supplemental_file.docx]

# Appendices

## Appendix A: Currency Codes for HRG Data Extraction

| **Currency** | **Currency description** |
| --- | --- |
| AA22C | Cerebrovascular Accident, Nervous System Infections or Encephalopathy, with CC Score 14+ |
| AA22D | Cerebrovascular Accident, Nervous System Infections or Encephalopathy, with CC Score 11-13 |
| AA22E | Cerebrovascular Accident, Nervous System Infections or Encephalopathy, with CC Score 8-10 |
| AA22F | Cerebrovascular Accident, Nervous System Infections or Encephalopathy, with CC Score 5-7 |
| AA22G | Cerebrovascular Accident, Nervous System Infections or Encephalopathy, with CC Score 0-4 |
| AA23C | Haemorrhagic Cerebrovascular Disorders with CC Score 14+ |
| AA23D | Haemorrhagic Cerebrovascular Disorders with CC Score 10-13 |
| AA23E | Haemorrhagic Cerebrovascular Disorders with CC Score 6-9 |
| AA23F | Haemorrhagic Cerebrovascular Disorders with CC Score 3-5 |
| AA23G | Haemorrhagic Cerebrovascular Disorders with CC Score 0-2 |
| AA35A | Stroke with CC Score 16+ |
| AA35B | Stroke with CC Score 13-15 |
| AA35C | Stroke with CC Score 10-12 |
| AA35D | Stroke with CC Score 7-9 |
| AA35E | Stroke with CC Score 4-6 |
| AA35F | Stroke with CC Score 0-3 |
| EB02A | Endocarditis with CC Score 10+ |
| EB02B | Endocarditis with CC Score 5-9 |
| EB02C | Endocarditis with CC Score 0-4 |
| EB03A | Heart Failure or Shock, with CC Score 14+ |
| EB03B | Heart Failure or Shock, with CC Score 11-13 |
| EB03C | Heart Failure or Shock, with CC Score 8-10 |
| EB03D | Heart Failure or Shock, with CC Score 4-7 |
| EB03E | Heart Failure or Shock, with CC Score 0-3 |
| EB04Z | Hypertension |
| EB05A | Cardiac Arrest with CC Score 9+ |
| EB05B | Cardiac Arrest with CC Score 5-8 |
| EB05C | Cardiac Arrest with CC Score 0-4 |
| EB06A | Cardiac Valve Disorders with CC Score 13+ |
| EB06B | Cardiac Valve Disorders with CC Score 9-12 |
| EB06C | Cardiac Valve Disorders with CC Score 5-8 |
| EB06D | Cardiac Valve Disorders with CC Score 0-4 |
| EB07A | Arrhythmia or Conduction Disorders, with CC Score 13+ |
| EB07B | Arrhythmia or Conduction Disorders, with CC Score 10-12 |
| EB07C | Arrhythmia or Conduction Disorders, with CC Score 7-9 |
| EB07D | Arrhythmia or Conduction Disorders, with CC Score 4-6 |
| EB07E | Arrhythmia or Conduction Disorders, with CC Score 0-3 |
| EB08A | Syncope or Collapse, with CC Score 13+ |
| EB08B | Syncope or Collapse, with CC Score 10-12 |
| EB08C | Syncope or Collapse, with CC Score 7-9 |

| EB08D | Syncope or Collapse, with CC Score 4-6 |
| --- | --- |
| EB08E | Syncope or Collapse, with CC Score 0-3 |
| EB09A | Non-Interventional Congenital Cardiac Conditions with CC Score 3+ |
| EB09B | Non-Interventional Congenital Cardiac Conditions with CC Score 0-2 |
| EB10A | Actual or Suspected Myocardial Infarction, with CC Score 13+ |
| EB10B | Actual or Suspected Myocardial Infarction, with CC Score 10-12 |
| EB10C | Actual or Suspected Myocardial Infarction, with CC Score 7-9 |
| EB10D | Actual or Suspected Myocardial Infarction, with CC Score 4-6 |
| EB10E | Actual or Suspected Myocardial Infarction, with CC Score 0-3 |
| EB12A | Unspecified Chest Pain with CC Score 11+ |
| EB12B | Unspecified Chest Pain with CC Score 5-10 |
| EB12C | Unspecified Chest Pain with CC Score 0-4 |
| EB13A | Angina with CC Score 12+ |
| EB13B | Angina with CC Score 8-11 |
| EB13C | Angina with CC Score 4-7 |
| EB13D | Angina with CC Score 0-3 |
| EB14A | Other Acquired Cardiac Conditions with CC Score 13+ |
| EB14B | Other Acquired Cardiac Conditions with CC Score 9-12 |
| EB14C | Other Acquired Cardiac Conditions with CC Score 6-8 |
| EB14D | Other Acquired Cardiac Conditions with CC Score 3-5 |
| EB14E | Other Acquired Cardiac Conditions with CC Score 0-2 |
| EB15A | Primary Pulmonary Hypertension with CC Score 9+ |
| EB15B | Primary Pulmonary Hypertension with CC Score 4-8 |
| EB15C | Primary Pulmonary Hypertension with CC Score 0-3 |
| EC10A | Very Complex Procedures for Congenital Heart Disease with CC Score 15+ |
| EC10B | Very Complex Procedures for Congenital Heart Disease with CC Score 7-14 |
| EC10C | Very Complex Procedures for Congenital Heart Disease with CC Score 0-6 |
| EC11A | Complex Procedures for Congenital Heart Disease with CC Score 15+ |
| EC11B | Complex Procedures for Congenital Heart Disease with CC Score 7-14 |
| EC11C | Complex Procedures for Congenital Heart Disease with CC Score 0-6 |
| EC12A | Very Major Procedures for Congenital Heart Disease with CC Score 9+ |
| EC12B | Very Major Procedures for Congenital Heart Disease with CC Score 4-8 |
| EC12C | Very Major Procedures for Congenital Heart Disease with CC Score 0-3 |
| EC13A | Major Procedures for Congenital Heart Disease with CC Score 9+ |
| EC13B | Major Procedures for Congenital Heart Disease with CC Score 4-8 |
| EC13C | Major Procedures for Congenital Heart Disease with CC Score 0-3 |
| EC14A | Intermediate Procedures for Congenital Heart Disease with CC Score 9+ |
| EC14B | Intermediate Procedures for Congenital Heart Disease with CC Score 4-8 |
| EC14C | Intermediate Procedures for Congenital Heart Disease with CC Score 0-3 |
| EC15A | Minor Procedures for Congenital Heart Disease with CC Score 4+ |
| EC15B | Minor Procedures for Congenital Heart Disease with CC Score 0-3 |
| EC20A | Diagnostic Percutaneous Intervention for Congenital Heart Disease with CC Score 4+ |
| EC20B | Diagnostic Percutaneous Intervention for Congenital Heart Disease with CC Score 0-3 |
| EC21Z | Complex Echocardiogram for Congenital Heart Disease |
| EC22Z | Electrocardiogram Monitoring or Stress Testing, for Congenital Heart Disease |
| ED01Z | Heart and Lung Transplant |
| ED04Z | Complex Heart Transplant |
| ED05Z | Standard Heart Transplant |
| ED06Z | Insertion of Short-Term Bridge to Transplant Ventricular Assist Device, with Heart Transplant |
| ED07Z | Insertion of Short-Term Bridge to Transplant Ventricular Assist Device, without Heart Transplant |
| ED08Z | Complex Insertion of Long-Term Bridge to Transplant Ventricular Assist Device |
| ED09Z | Standard Insertion of Long-Term Bridge to Transplant Ventricular Assist Device |
| ED11Z | Standard Repair of Descending Thoracic Aorta |
| ED12A | Complex Repair of Aortic Root with CC Score 7+ |
| ED12B | Complex Repair of Aortic Root with CC Score 0-6 |
| ED13A | Standard Repair of Aortic Root with CC Score 7+ |
| ED13B | Standard Repair of Aortic Root with CC Score 0-6 |
| ED14A | Complex Repair of Ascending Thoracic Aorta with CC Score 7+ |
| ED14B | Complex Repair of Ascending Thoracic Aorta with CC Score 0-6 |
| ED15A | Standard Repair of Ascending Thoracic Aorta with CC Score 7+ |
| ED15B | Standard Repair of Ascending Thoracic Aorta with CC Score 0-6 |
| ED16Z | Hybrid Repair of Descending Thoracic Aorta or Aortic Arch |
| ED17Z | Very Complex Repair of Descending Thoracic Aorta or Aortic Arch |
| ED18Z | Complex Repair of Descending Thoracic Aorta or Aortic Arch |
| ED20A | Complex, Repair or Replacement, of Multiple Heart Valves, with CC Score 8+ |
| ED20B | Complex, Repair or Replacement, of Multiple Heart Valves, with CC Score 0-7 |
| ED21A | Standard, Repair or Replacement, of Multiple Heart Valves, with CC Score 8+ |
| ED21B | Standard, Repair or Replacement, of Multiple Heart Valves, with CC Score 0-7 |
| ED22A | Complex, Coronary Artery Bypass Graft with Single Heart Valve Replacement or Repair, with CC Score 11+ |
| ED22B | Complex, Coronary Artery Bypass Graft with Single Heart Valve Replacement or Repair, with CC Score 6-10 |
| ED22C | Complex, Coronary Artery Bypass Graft with Single Heart Valve Replacement or Repair, with CC Score 0-5 |
| ED23A | Standard, Coronary Artery Bypass Graft with Single Heart Valve Replacement or Repair, with CC Score 11+ |
| ED23B | Standard, Coronary Artery Bypass Graft with Single Heart Valve Replacement or Repair, with CC Score 6-10 |
| ED23C | Standard, Coronary Artery Bypass Graft with Single Heart Valve Replacement or Repair, with CC Score 0-5 |
| ED24A | Complex, Single Heart Valve Replacement or Repair, with CC Score 11+ |
| ED24B | Complex, Single Heart Valve Replacement or Repair, with CC Score 6-10 |
| ED24C | Complex, Single Heart Valve Replacement or Repair, with CC Score 0-5 |
| ED25A | Standard, Single Heart Valve Replacement or Repair, with CC Score 11+ |
| ED25B | Standard, Single Heart Valve Replacement or Repair, with CC Score 6-10 |
| ED25C | Standard, Single Heart Valve Replacement or Repair, with CC Score 0-5 |
| ED26A | Complex Coronary Artery Bypass Graft with CC Score 10+ |
| ED26B | Complex Coronary Artery Bypass Graft with CC Score 5-9 |
| ED26C | Complex Coronary Artery Bypass Graft with CC Score 0-4 |
| ED27A | Major Coronary Artery Bypass Graft with CC Score 10+ |
| ED27B | Major Coronary Artery Bypass Graft with CC Score 5-9 |
| ED27C | Major Coronary Artery Bypass Graft with CC Score 0-4 |
| ED28A | Standard Coronary Artery Bypass Graft with CC Score 10+ |
| ED28B | Standard Coronary Artery Bypass Graft with CC Score 5-9 |
| ED28C | Standard Coronary Artery Bypass Graft with CC Score 0-4 |
| ED30A | Complex, Other Operations on Heart or Pericardium, with CC Score 10+ |
| ED30B | Complex, Other Operations on Heart or Pericardium, with CC Score 5-9 |
| ED30C | Complex, Other Operations on Heart or Pericardium, with CC Score 0-4 |
| ED31A | Standard, Other Operations on Heart or Pericardium, with CC Score 10+ |
| ED31B | Standard, Other Operations on Heart or Pericardium, with CC Score 5-9 |
| ED31C | Standard, Other Operations on Heart or Pericardium, with CC Score 0-4 |
| EY01A | Implantation of Cardioverter Defibrillator with Cardiac Resynchronisation Therapy, with CC Score 9+ |
| EY01B | Implantation of Cardioverter Defibrillator with Cardiac Resynchronisation Therapy, with CC Score 0-8 |
| EY02A | Implantation of Cardioverter Defibrillator with CC Score 9+ |
| EY02B | Implantation of Cardioverter Defibrillator with CC Score 0-8 |
| EY03Z | Implantation of Biventricular Pacemaker with Other Percutaneous Intervention |
| EY04A | Implantation of Biventricular Pacemaker with CC Score 6+ |
| EY04B | Implantation of Biventricular Pacemaker with CC Score 0-5 |
| EY05A | Implantation of Dual-Chamber Pacemaker with Other Percutaneous Intervention, with CC Score 6+ |
| EY05B | Implantation of Dual-Chamber Pacemaker with Other Percutaneous Intervention, with CC Score 0-5 |
| EY06A | Implantation of Dual-Chamber Pacemaker with CC Score 12+ |
| EY06B | Implantation of Dual-Chamber Pacemaker with CC Score 9-11 |
| EY06C | Implantation of Dual-Chamber Pacemaker with CC Score 6-8 |
| EY06D | Implantation of Dual-Chamber Pacemaker with CC Score 3-5 |
| EY06E | Implantation of Dual-Chamber Pacemaker with CC Score 0-2 |
| EY07A | Implantation of Single-Chamber Pacemaker with Other Percutaneous Intervention, with CC Score 6+ |
| EY07B | Implantation of Single-Chamber Pacemaker with Other Percutaneous Intervention, with CC Score 0-5 |
| EY08A | Implantation of Single-Chamber Pacemaker with CC Score 12+ |
| EY08B | Implantation of Single-Chamber Pacemaker with CC Score 9-11 |
| EY08C | Implantation of Single-Chamber Pacemaker with CC Score 6-8 |
| EY08D | Implantation of Single-Chamber Pacemaker with CC Score 3-5 |
| EY08E | Implantation of Single-Chamber Pacemaker with CC Score 0-2 |
| EY11Z | Testing of Cardiac Pacemaker or Cardioverter Defibrillator |
| EY12A | Implantation of Electrocardiography Loop Recorder with CC Score 3+ |
| EY12B | Implantation of Electrocardiography Loop Recorder with CC Score 0-2 |
| EY13Z | Removal of Electrocardiography Loop Recorder |
| EY14A | Implantation of Cardioverter Defibrillator with Cardiac Resynchronisation Therapy, with Extraction or Major Open Procedure, with CC Score 9+ |
| EY14B | Implantation of Cardioverter Defibrillator with Cardiac Resynchronisation Therapy, with Extraction or Major Open Procedure, with CC Score 0-8 |
| EY15A | Implantation of Cardioverter Defibrillator, with Extraction or Major Open Procedure, with CC Score 9+ |
| EY15B | Implantation of Cardioverter Defibrillator, with Extraction or Major Open Procedure, with CC Score 0-8 |
| EY16A | Extraction of Cardiac Pacemaker or Cardioverter Defibrillator, with CC Score 10+ |
| EY16B | Extraction of Cardiac Pacemaker or Cardioverter Defibrillator, with CC Score 0-9 |
| EY17A | Explantation or Attention to, Cardiac Pacemaker or Cardioverter Defibrillator, with CC Score 6+ |
| EY17B | Explantation or Attention to, Cardiac Pacemaker or Cardioverter Defibrillator, with CC Score 0-5 |
| EY20A | Transcatheter Aortic Valve Implantation (TAVI) using Other Approach, with CC Score 8+ |
| EY20B | Transcatheter Aortic Valve Implantation (TAVI) using Other Approach, with CC Score 0-7 |
| EY21A | Transcatheter Aortic Valve Implantation (TAVI) using Transfemoral Approach, with CC Score 8+ |
| EY21B | Transcatheter Aortic Valve Implantation (TAVI) using Transfemoral Approach, with CC Score 0-7 |
| EY22A | Complex Other Percutaneous Transluminal Repair of Acquired Defect of Heart with CC Score 10+ |
| EY22B | Complex Other Percutaneous Transluminal Repair of Acquired Defect of Heart with CC Score 5-9 |
| EY22C | Complex Other Percutaneous Transluminal Repair of Acquired Defect of Heart with CC Score 0-4 |
| EY23A | Standard Other Percutaneous Transluminal Repair of Acquired Defect of Heart with CC Score 10+ |
| EY23B | Standard Other Percutaneous Transluminal Repair of Acquired Defect of Heart with CC Score 5-9 |
| EY23C | Standard Other Percutaneous Transluminal Repair of Acquired Defect of Heart with CC Score 0-4 |
| EY30A | Complex Percutaneous Transluminal Ablation of Heart with CC Score 3+ |
| EY30B | Complex Percutaneous Transluminal Ablation of Heart with CC Score 0-2 |
| EY31A | Standard Percutaneous Transluminal Ablation of Heart with CC Score 3+ |
| EY31B | Standard Percutaneous Transluminal Ablation of Heart with CC Score 0-2 |
| EY32A | Percutaneous Diagnostic Electrophysiology Studies with CC Score 2+ |
| EY32B | Percutaneous Diagnostic Electrophysiology Studies with CC Score 0-1 |
| EY40A | Complex Percutaneous Transluminal Coronary Angioplasty with CC Score 12+ |
| EY40B | Complex Percutaneous Transluminal Coronary Angioplasty with CC Score 8-11 |
| EY40C | Complex Percutaneous Transluminal Coronary Angioplasty with CC Score 4-7 |
| EY40D | Complex Percutaneous Transluminal Coronary Angioplasty with CC Score 0-3 |
| EY41A | Standard Percutaneous Transluminal Coronary Angioplasty with CC Score 12+ |
| EY41B | Standard Percutaneous Transluminal Coronary Angioplasty with CC Score 8-11 |
| EY41C | Standard Percutaneous Transluminal Coronary Angioplasty with CC Score 4-7 |
| EY41D | Standard Percutaneous Transluminal Coronary Angioplasty with CC Score 0-3 |
| EY42A | Complex Cardiac Catheterisation with CC Score 7+ |
| EY42B | Complex Cardiac Catheterisation with CC Score 4-6 |
| EY42C | Complex Cardiac Catheterisation with CC Score 2-3 |
| EY42D | Complex Cardiac Catheterisation with CC Score 0-1 |
| EY43A | Standard Cardiac Catheterisation with CC Score 13+ |
| EY43B | Standard Cardiac Catheterisation with CC Score 10-12 |
| EY43C | Standard Cardiac Catheterisation with CC Score 7-9 |
| EY43D | Standard Cardiac Catheterisation with CC Score 4-6 |
| EY43E | Standard Cardiac Catheterisation with CC Score 2-3 |
| EY43F | Standard Cardiac Catheterisation with CC Score 0-1 |
| EY44A | Very Complex Percutaneous Transluminal Coronary Angioplasty with CC Score 12+ |
| EY44B | Very Complex Percutaneous Transluminal Coronary Angioplasty with CC Score 8-11 |
| EY44C | Very Complex Percutaneous Transluminal Coronary Angioplasty with CC Score 4-7 |
| EY44D | Very Complex Percutaneous Transluminal Coronary Angioplasty with CC Score 0-3 |
| EY50Z | Complex Echocardiogram |
| EY51Z | Electrocardiogram Monitoring or Stress Testing |
| PE23A | Paediatric Cardiac Conditions with CC Score 13+ |
| PE23B | Paediatric Cardiac Conditions with CC Score 10-12 |
| PE23C | Paediatric Cardiac Conditions with CC Score 6-9 |
| PE23D | Paediatric Cardiac Conditions with CC Score 3-5 |
| PE23E | Paediatric Cardiac Conditions with CC Score 1-2 |
| PE23F | Paediatric Cardiac Conditions with CC Score 0 |
| RD08Z | Cardiac Magnetic Resonance Imaging Scan without Contrast |
| RD09Z | Cardiac Magnetic Resonance Imaging Scan with Post-Contrast Only |
| RD10Z | Cardiac Magnetic Resonance Imaging Scan with Pre- and Post-Contrast |
| RD47Z | Vascular Ultrasound Scan |
| RD47Z | Vascular Ultrasound Scan |
| RD60Z | Cardiac Computerised Tomography Scan |
| VC04Z | Rehabilitation for Stroke |
| VC38Z | Rehabilitation for Acute Myocardial Infarction or Other Cardiac Disorders |
| YG10Z | Percutaneous Transvascular Biopsy of Lesion of Liver |
| YG10Z | Percutaneous Transvascular Biopsy of Lesion of Liver |
| YQ50A | Peripheral Vascular Disorders with CC Score 15+ |
| YQ50A | Peripheral Vascular Disorders with CC Score 15+ |
| YQ50B | Peripheral Vascular Disorders with CC Score 11-14 |
| YQ50B | Peripheral Vascular Disorders with CC Score 11-14 |
| YQ50C | Peripheral Vascular Disorders with CC Score 8-10 |
| YQ50C | Peripheral Vascular Disorders with CC Score 8-10 |
| YQ50D | Peripheral Vascular Disorders with CC Score 5-7 |
| YQ50D | Peripheral Vascular Disorders with CC Score 5-7 |
| YQ50E | Peripheral Vascular Disorders with CC Score 2-4 |
| YQ50E | Peripheral Vascular Disorders with CC Score 2-4 |
| YQ50F | Peripheral Vascular Disorders with CC Score 0-1 |
| YQ50F | Peripheral Vascular Disorders with CC Score 0-1 |
| YR60Z | Complex Endovascular Repair of Thoracoabdominal Aortic Aneurysm using Branched Stent Graft |
| YR61Z | Standard Endovascular Repair of Thoracoabdominal Aortic Aneurysm using Branched Stent Graft |
| YR62A | Complex Endovascular Repair of Thoracoabdominal Aortic Aneurysm using Fenestrated Stent Graft, with CC Score 6+ |
| YR62B | Complex Endovascular Repair of Thoracoabdominal Aortic Aneurysm using Fenestrated Stent Graft, with CC Score 0-5 |
| YR63A | Standard Endovascular Repair of Thoracoabdominal Aortic Aneurysm using Fenestrated Stent Graft, with CC Score 6+ |
| YR63B | Standard Endovascular Repair of Thoracoabdominal Aortic Aneurysm using Fenestrated Stent Graft, with CC Score 0-5 |
| YR64A | Complex Endovascular Repair of Thoracic Aortic Aneurysm, with CC Score 6+ |
| YR64B | Complex Endovascular Repair of Thoracic Aortic Aneurysm, with CC Score 0-5 |
| YR65A | Standard Endovascular Repair of Thoracic Aortic Aneurysm, with CC Score 6+ |
| YR65B | Standard Endovascular Repair of Thoracic Aortic Aneurysm, with CC Score 0-5 |
| YR66A | Complex Endovascular Repair of Abdominal Aortic Aneurysm, with CC Score 6+ |
| YR66B | Complex Endovascular Repair of Abdominal Aortic Aneurysm, with CC Score 0-5 |
| YR67A | Standard Endovascular Repair of Abdominal Aortic Aneurysm, with CC Score 6+ |
| YR67B | Standard Endovascular Repair of Abdominal Aortic Aneurysm, with CC Score 0-5 |

## Appendix B: Outpatient Clinic Tallies

### NHS England

| Appendix B1. Activity levels, unit costs, and total costs of outpatient services in NHS England for the 2019-2022 fiscal years. *Amounts have been adjusted by the proportion of consultations that are expected to have been from CVD for generalist specialties. All figures are in nominal terms. | | | | | | | | | |
| --- | --- | --- | --- | --- | --- | --- | --- | --- | --- |
|  | **2019/20** | | | **2020/21** | | | **2021/22** | | |
| **Service description** | **Activity** | **Unit Cost** | **Total Cost** | **Activity** | **Unit Cost** | **Total Cost** | **Activity** | **Unit Cost** | **Total Cost** |
| Vascular Surgery | 504,771 | £ 160 | £ 80,744,241 | 368,523 | £ 230 | £ 84,758,066 | 440,254 | £ 207 | £ 91,307,646 |
| Cardiothoracic Surgery | 44,956 | £ 212 | £ 9,541,028 | 33,184 | £ 327 | £ 10,854,297 | 40,233 | £ 401 | £ 16,150,219 |
| Cardiac Surgery | 74,392 | £ 272 | £ 20,242,990 | 60,070 | £ 321 | £ 19,302,146 | 70,322 | £ 332 | £ 23,350,998 |
| Cardiothoracic Transplantation | 15,128 | £ 371 | £ 5,614,258 | 14,507 | £ 434 | £ 6,290,933 | 14,112 | £ 495 | £ 6,984,992 |
| Paediatric Cardiac Surgery | 1,891 | £ 457 | £ 864,890 | 2,051 | £ 397 | £ 813,518 | 2,071 | £ 476 | £ 985,840 |
| General Medicine | 934,731 | £ 178 | £ 29,720,04* | 821,675 | £ 211 | £ 172,980,286 | 1,116,502 | £ 153 | £ 170,779,688 |
| Paediatric Cardiology | 147,905 | £ 176 | £ 26,093,650 | 2,444,657 | £ 191 | £ 467,226,530 | 2,951,106 | £ 169 | £ 499,890,209 |
| Anticoagulant Service | 1,649,817 | £ 43 | £ 71,043,140 | 137,538 | £ 244 | £ 33,590,444 | 162,217 | £ 227 | £ 36,888,532 |
| Cardiac Rehabilitation | 132,254 | £ 106 | £ 14,046,224 | 1,245,227 | £ 60 | £ 75,168,774 | 105,830 | £ 134 | £ 14,196,405 |
| Stroke Medicine | 83,928 | £ 224 | £ 18,759,297 | 82,196 | £ 159 | £ 13,088,571 | 101,630 | £ 302 | £ 30,706,494 |
| Transient Ischaemic Attack | 105,057 | £ 191 | £ 20,028,440 | 85,014 | £ 288 | £ 24,520,810 | 85,094 | £ 293 | £ 24,914,531 |
| Congenital Heart Disease Service | 28,742 | £ 158 | £ 4,527,102 | 76,116 | £ 306 | £ 23,260,395 | 25,191 | £ 267 | £ 6,729,712 |
| Geriatric Medicine | 370,795 | £ 270 | £ 17,900,168 | 24,268 | £ 240 | £ 5,818,202 | 299,995 | £ 372 | £ 111,637,210 |
| Cardiology | 2,888,284 | £ 142 | £ 410,047,893* | 271,024 | £ 401 | £ 108,785,131 | 105,830 | £ 134 | £ 14,196,405 |
| Acute Internal Medicine |  |  |  |  |  |  | 30,390 | £ 82 | £ 2,496,470 |
| **Total** | **5,910,223.58** |  | **£ 729,173,360** | 4,768,449.82 |  | £ 815,001,234 | 5,412,187.25 |  | £ 883,921,864 |

### NHS Scotland

| Appendix B2. Activity levels, unit costs, and total costs of outpatient services in NHS Scotland for the 2019-2022 fiscal years. *Amounts have been adjusted by the proportion of consultations that are expected to have been from CVD for generalist specialties. | | | | | | | | | |
| --- | --- | --- | --- | --- | --- | --- | --- | --- | --- |
|  | **2019/20** | | | **2020/21** | | | **2021/22** | | |
| **Service description** | **Activity** | **Unit Costs** | **Total Cost** | **Activity** | **Unit Costs** | **Total Cost** | **Activity** | **Unit Costs** | **Total Cost** |
| Cardiology | 105,610.00 | £ 141.97 | £ 14,993,386.37 | 78,623.00 | £ 191.12 | £15,026,546.25 | 91,776.00 | £ 169.39 | £ 15,546,010.15 |
| Cardiothoracic Surgery | 11,550.00 | £ 272.11 | £ 3,142,898.94 | 9,990.00 | £ 321.33 | £ 3,210,062.22 | 12,426.00 | £ 332.06 | £4,126,155.37 |
| General Medicine | 68,937.00 | £ 178.08 | £ 2,191,286.92* | 52,956.00 | £ 210.52 | £ 1,989,985.58 | 69,846.00 | £ 152.96 | £1,907,025.37 |
| General Surgery | 413,226.00 | £ 159.96 | £ 14,963,362.36* | 269,021.00 | £ 229.99 | £11,425,225.50 | 354,530.00 | £ 207.40 | £ 13,857,452.37 |
| Geriatric Medicine | 40,346.00 | £ 270.38 | £ 1,947,187.75* | 27,665.00 | £ 401.39 | £ 1,982,123.38 | 33,170.00 | £ 372.13 | £2,203,325.45 |
| Neurology | 95,662.00 | £ 205.24 | £ 2,335,313.83* | 86,664.00 | £ 296.54 | £ 2,661,503.16 | 95,360.00 | £ 297.88 | £3,176,568.30 |
| Paediatric Surgery | 16,117.00 | £ 457.37 | £ 17,587.23 | 10,141.00 | £ 396.64 | £ 12,357.73 | 13,208.00 | £ 476.02 | £15,894.35 |
| Paediatrics | 117,079.00 | £ 173.34 | £ 2,014,044.20* | 104,458.00 | £ 243.55 | £ 2,467,651.35 | 115,784.00 | £ 232.74 | £2,751,917.76 |
| **Total** | **868,527.00** |  | **£ 41,605,067.60** | 639,518.00 |  | £38,775,455.18 | 786,100.00 |  | £ 43,584,349.13 |

### NHS Northern Ireland

| Appendix B3. Activity levels, unit costs, and total costs of outpatient services in NHS Northern Ireland for the 2019-2022 fiscal years. *Amounts have been adjusted by the proportion of consultations that are expected to have been from CVD for generalist specialties. | | | | | | | | | |
| --- | --- | --- | --- | --- | --- | --- | --- | --- | --- |
|  | **2019/20** | | | **2020/21** | | | **2021/22** | | |
| **Specialty** | **Activity** | **Unit Cost** | **Total Cost** | **Activity** | **Unit Cost** | **Total Cost** | **Activity** | **Unit Cost** | **Total Cost** |
| Cardiac Surgery | 2,413 | £ 272.11 | £ 656,607.37 | 936 | £321.33 | £ 300,762.59 | 1372 | £332.06 | £ 455,583.87 |
| Cardiology | 73,808 | £ 141.97 | £ 10,478,476.10 | 28,497 | £191.12 | £5,446,389.59 | 35,718 | £169.39 | £6,050,300.63 |
| General Medicine | 63,270 | £ 178.08 | £ 2,011,151.10* | 25,351 | £210.52 | £ 952,642.28 | 44,697 | £152.96 | £1,220,375.01 |
| General Surgery | 165,575 | £ 159.96 | £ 5,995,650.62* | 74,366 | £229.99 | £3,158,297.38 | 101,751 | £207.40 | £3,977,123.62 |
| Geriatric Medicine | 32,997 | £ 270.38 | £ 1,592,508.66* | 12,235 | £401.39 | £ 876,605.08 | 20,094 | £372.13 | £1,334,748.92 |
| Neurology | 41,805 | £ 205.24 | £ 1,020,549.38 | 14,325 | £296.54 | £ 439,929.30 | 23,049 | £297.88 | £ 767,792.81 |
| Paediatric Surgery | 7,212 | £ 457.37 | £ 7,869.90 | 3,923 | £396.64 | £4,780.53 | 5,843 | £476.02 | £7,031.40 |
| Paediatrics | 126,038 | £ 173.34 | £ 2,168,160.84* | 77,868 | £243.55 | £1,839,505.60 | 108,593 | £232.74 | £2,581,004.33 |
| **Total** | **513,118** |  | **£ 23,930,973.96** | 237,501 |  | £ 13,018,912.34 |  |  | £ 16,393,960.59 |

## Appendix C: Codes used to identify CVD-related A&E Consultations in England

| Appendix C. SNOMEDCT Codes used for the identification of CVD-related A&E attendances in NHS England. | |
| --- | --- |
| **SNOMEDCT** | **SNOMEDCT Description** |
| 10392004 | Injury of abdominal aorta (disorder) |
| 126736007 | Neoplasm of blood vessel (disorder) |
| 128053003 | Deep venous thrombosis (disorder) |
| 13213009 | Congenital heart disease (disorder) |
| 192759008 | Cerebral venous sinus thrombosis (disorder) |
| 195060002 | Ventricular pre-excitation (disorder) |
| 230508004 | Arteritic ischemic optic neuropathy (disorder) |
| 230690007 | Cerebrovascular accident (disorder) |
| 230730001 | Dissection of vertebral artery (disorder) |
| 233819005 | Stable angina (disorder) |
| 233981004 | Arterial aneurysm (disorder) |
| 233985008 | Abdominal aortic aneurysm (disorder) |
| 234000009 | Dissection of femoral artery (disorder) |
| 25569003 | Ventricular tachycardia (disorder) |
| 266257000 | Transient ischemic attack (disorder) |
| 266262004 | Arterial embolus and thrombosis (disorder) |
| 270492004 | First degree atrioventricular block (disorder) |
| 271966006 | Disorder of cardiovascular prostheses and implants (disorder) |
| 27885002 | Complete atrioventricular block (disorder) |
| 28189009 | Mobitz type II atrioventricular block (disorder) |
| 297157005 | Intracranial venous thrombosis (disorder) |
| 29717002 | Premature beats (disorder) |
| 307322002 | Rupture radial artery (disorder) |
| 308546005 | Dissection of aorta (disorder) |
| 3238004 | Pericarditis (disorder) |
| 36083008 | Sick sinus syndrome (disorder) |
| 367363000 | Right ventricular failure (disorder) |
| 368009 | Heart valve disorder (disorder) |
| 373945007 | Pericardial effusion (disorder) |
| 38341003 | Hypertensive disorder, systemic arterial (disorder) |
| 38742007 | Central retinal artery occlusion (disorder) |
| 387840005 | Neoplasm of heart AND/OR pericardium (disorder) |
| 394659003 | Acute coronary syndrome (disorder) |
| 400047006 | Peripheral vascular disease (disorder) |
| 401303003 | Acute ST segment elevation myocardial infarction (disorder) |
| 401314000 | Acute non-ST segment elevation myocardial infarction (disorder) |
| 402863005 | Venous stasis ulcer of leg (disorder) |
| 40541001 | Acute pulmonary edema (disorder) |
| 410429000 | Cardiac arrest (disorder) |
| 418341009 | Atrioventricular conduction disorder (disorder) |
| 42343007 | Congestive heart failure (disorder) |
| 429257001 | Disorder of transplanted heart (disorder) |
| 431415002 | Management of internal defibrillation (procedure) |
| 433068007 | Aneurysm of thoracic aorta (disorder) |
| 44808001 | Conduction disorder of the heart (disorder) |
| 4557003 | Preinfarction syndrome (disorder) |
| 49436004 | Atrial fibrillation (disorder) |
| 49982000 | Multifocal atrial tachycardia (disorder) |
| 50821009 | Arterial retinal branch occlusion (disorder) |
| 50920009 | Myocarditis (disorder) |
| 54016002 | Mobitz type I incomplete atrioventricular block (disorder) |
| 56819008 | Endocarditis (disorder) |
| 59118001 | Right bundle branch block (disorder) |
| 59282003 | Pulmonary embolism (disorder) |
| 63467002 | Left bundle branch block (disorder) |
| 6456007 | Supraventricular tachycardia (disorder) |
| 6962006 | Hypertensive retinopathy (disorder) |
| 70272006 | Malignant hypertension (disorder) |
| 70995007 | Pulmonary hypertension (disorder) |
| 720626009 | Dissection of carotid artery (disorder) |
| 72866009 | Varicose veins of lower extremity (disorder) |
| 74021003 | Bifascicular block (disorder) |
| 84114007 | Heart failure (disorder) |
| 85232009 | Left heart failure (disorder) |
| 85898001 | Cardiomyopathy (disorder) |
| 86014007 | Trifascicular block (disorder) |
| 86175003 | Injury of heart (disorder) |
| 872381000000101 | Arterial lower limb ulcer (disorder) |
| 88032003 | Amaurosis fugax (disorder) |
| 89138009 | Cardiogenic shock (disorder) |
| 91489000 | Acute vascular insufficiency of intestine (disorder) |
| 9651007 | Long QT syndrome (disorder) |

## Appendix D: Stroke LTC Markov Model

### Appendix D1: Markov Model


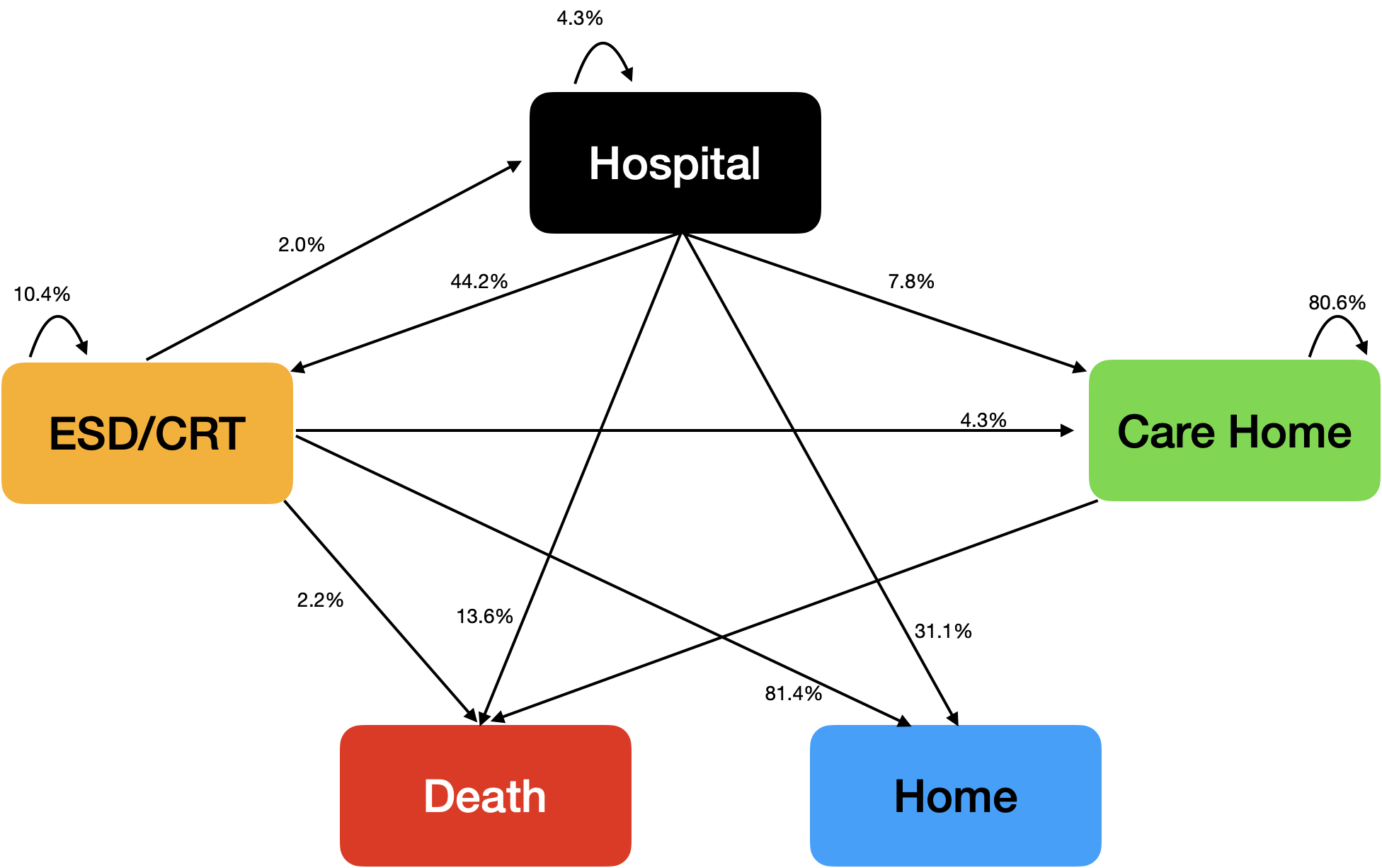


Appendix D1. Markov model for Stroke-related LTC with transition probabilities between each state.

### Appendix D2: Transition Probabilities and Costs

| Appendix D2. Transition probabilities and statistics used in the Markov model for Stroke LTC care. SEM – standard error of the mean | | | | | |
| --- | --- | --- | --- | --- | --- |
| **Transition Probabilities** | **Probability** | **95% Lower** | **95% Upper** | **SEM** | **Distribution** |
| Hospital to Care Home | 0.078358747 | 0.072633334 | 0.078358747 | 0.00146059 | Beta |
| Hospital to Death | 0.136078028 | 0.134042287 | 0.136078028 | 0.00141313 | Beta |
| Hospital to Home or Other | 0.311141925 | 0.306388038 | 0.311141925 | 0.00121275 | Beta |
| Hospital to Early Supported Discharge (ESD) | 0.441894447 | 0.437696396 | 0.441894447 | 0.00107095 | Beta |
| Hospital to Hospital | 0.044 | 0.037967244 | 0.043799554 | 0.00148786 | Beta |
| ESD to Care Home | 0.043355289 | 0.041366163 | 0.0434 | 0.00246548 | Beta |
| ESD to Death | 0.022293861 | 0.020863718 | 0.022293861 | 0.00248364 | Beta |
| ESD to Home or Other | 0.814564305 | 0.810901855 | 0.814564305 | 0.00103638 | Beta |
| ESD to Hospital | 0.020181709 | 0.019699209 | 0.020181709 | 0.00087167 | Beta |
| ESD to ESD | 0.104470589 | 0.102303302 | 0.104470589 | 0.00171951 | Beta |
| Care Home to Death | 0.193902036 | 0.189521123 | 0.198267285 | 0.00223116 | Beta |
| Care Home to Care Home | 0.806097964 |  |  |  |  |
|  | **Cost** |  |  |  |  |
| Care Home Cost per Patient per Year (adjusted for deaths) | £ 61,745.61 |  |  |  |  |

##

Appendix E: Medical Device Costs

| Appendix E. NHS England Medical Devices included in the high-cost device program, including activity, unit costs, and total costs, in 2019-2022. | | | | | | | | | | | |
| --- | --- | --- | --- | --- | --- | --- | --- | --- | --- | --- | --- |
|  | 2019/2020 | | | | 2020/2021 | | | 2021/2022 | | | |
| **Description** | **Activity** | **Avg Unit Cost** | **Total Costs** | **Activity** | | **Avg Unit Cost** | **Total Costs** | | **Activity** | **Avg Unit Cost** | **Total Costs** |
| 3 dimensional mapping and linear ablation catheters used for complex cardiac ablation procedures | 52714 | £559.39 | £29,487,460.72 | 9973 | | £2,016.98 | £20,115,334.38 | | 18730 | £1,966.39 | £36,830,576.75 |
| Aneurysm coils (Separated aneurysm coils and flow diverters for intracranial aneurysms) | 11097 | £2,070.52 | £22,976,614.38 | 5488 | | £4,313.36 | £23,671,742.26 | | 7199 | £4,414.04 | £31,776,645.36 |
| Carotid, iliac and renal stents (Includes embolic protection devices) | 7567 | £708.00 | £5,357,411.75 | 5412 | | £859.45 | £4,651,344.48 | | 5160 | £931.50 | £4,806,550.61 |
| Devices used in connection with pulmonary artery banding | 306 | £8,014.59 | £2,452,466.03 | 383 | | £7,517.78 | £2,879,308.03 | | 927 | £8,688.62 | £8,054,350.69 |
| Drug-eluting peripheral angioplasty balloon | 3010 | £1,139.92 | £3,431,169.91 | 3067 | | £719.94 | £2,208,053.11 | | 4340 | £1,708.52 | £7,414,963.61 |
| Endovascular stent graft (Includes aortic stent grafts) | 7353 | £5,573.11 | £40,979,054.31 | 6164 | | £3,828.42 | £23,598,405.51 | | 7621 | £3,928.48 | £29,938,978.20 |
| Flow diverters for intracranial aneurysms | 828 | £2,553.49 | £2,114,286.14 | 559 | | £2,808.72 | £1,570,073.03 | | 580 | £4,216.60 | £2,445,626.44 |
| ICD (Implantable Cardioverter-Defibrillator) | 5341 | £9,589.46 | £51,217,326.64 | 4917 | | £7,986.93 | £39,271,751.42 | | 6445 | £8,370.16 | £53,945,680.29 |
| ICD with CRT (Cardiac Resynchronisation Therapy) capability | 2605 | £7,640.82 | £19,904,345.62 | 2554 | | £7,107.15 | £18,151,658.25 | | 3090 | £10,540.39 | £32,569,808.56 |
| Intracardiac pacemaker system | 6733 | £1,089.43 | £7,335,137.47 | 5400 | | £1,181.74 | £6,381,405.18 | | 5673 | £1,372.83 | £7,788,088.04 |
| Intracranial stents | 1942 | £1,431.75 | £2,780,457.00 | 2440 | | £1,751.91 | £4,274,662.87 | | 1912 | £2,066.74 | £3,951,608.03 |
| Occluder, vascular, appendage and septal devices | 6978 | £1,030.48 | £7,190,689.09 | 5662 | | £919.81 | £5,207,955.45 | | 6725 | £1,102.64 | £7,415,231.14 |
| Percutaneous valve repair and replacement devices (includes devices for TAVI) | 5621 | £5,989.08 | £33,664,631.12 | 4963 | | £8,539.84 | £42,383,223.46 | | 7717 | £12,682.53 | £97,871,094.42 |
| Peripheral vascular stents (Includes peripheral vascular drug eluting stents) | 12958 | £997.19 | £12,921,583.92 | 10146 | | £912.04 | £9,253,549.80 | | 12302 | £985.90 | £12,128,533.12 |
| Wireless CRT-P (Cardiac Resynchronisation Therapy-Pacemaker) system | 929 | £2,748.54 | £2,553,392.72 | 1122 | | £2,564.88 | £2,877,792.17 | | 1801 | £1,927.31 | £3,471,090.21 |

##

## Appendix F: Mortality Model

| Appendix F. Variables for used for the mortality model. SEM – standard error of the mean. | | | | |
| --- | --- | --- | --- | --- |
| **Employment Rate Category** | **Employment Rate** | **SEM** | **Distribution** |  |
| Male Employment 15-17 | 0.219 | 0.00306122 | *Beta* |  |
| Male Employment 18-24 | 0.618 | 0.00306122 | *Beta* |  |
| Male Employment 25-34 | 0.903 | 0.00306122 | *Beta* |  |
| Male Employment 35-49 | 0.916 | 0.00306122 | *Beta* |  |
| Male Employment 50-64 | 0.768 | 0.00306122 | *Beta* |  |
| Male Employment 65+ | 0.150 | 0.00306122 | *Beta* |  |
| Female Employment 15-17 | 0.277 | 0.00306122 | *Beta* |  |
| Female Employment 18-24 | 0.604 | 0.00306122 | *Beta* |  |
| Female Employment 25-34 | 0.79 | 0.00306122 | *Beta* |  |
| Female Employment 35-49 | 0.797 | 0.00306122 | *Beta* |  |
| Female Employment 50-64 | 0.684 | 0.00306122 | *Beta* |  |
| Female Employment 65+ | 0.088 | 0.00306122 | *Beta* |  |
|  |  |  |  |  |
| **Salary Category** | **Annual Salary (£)** | **SEM** | **Distribution** |  |
| Male Salary 15-17 | £5,766 | 691.92 | *Gamma* |  |
| Male Salary 18-21 | £13,929 | 487.515 | *Gamma* |  |
| Male Salary 22-29 | £26,975 | 296.725 | *Gamma* |  |
| Male Salary 30-39 | £37,792 | 377.92 | *Gamma* |  |
| Male Salary 40-49 | £44,673 | 536.076 | *Gamma* |  |
| Male Salary 50-59 | £42,688 | 554.944 | *Gamma* |  |
| Male Salary 60-65 | £31,511 | 598.709 | *Gamma* |  |
| Female Salary 15-18 | £3,600 | 316.8 | *Gamma* |  |
| Female Salary 19-21 | £10,134 | 324.288 | *Gamma* |  |
| Female Salary 22-29 | £21,365 | 192.285 | *Gamma* |  |
| Female Salary 30-39 | £25,752 | 206.016 | *Gamma* |  |
| Female Salary 40-49 | £26,952 | 269.52 | *Gamma* |  |
| Female Salary 50-59 | £24,500 | 245 | *Gamma* |  |
| Female Salary 60-65 | £17,640 | 1,110 | *Gamma* |  |

## Appendix G. One-way Sensitivity Analysis

| Appendix G. One-way sensitivity analysis variables used. 95% CI – 95% confidence interval. LL – lower limit. UL – upper limit. VaD – Vascular Dementia | | | |
| --- | --- | --- | --- |
|  | **Base Case** | **Lower Limit** | **Upper Limit** |
| **Primary Care** |  |  |  |
| Proportion of Consultations as CVD Visits | 0.12 | 0.09 | 0.15 |
| **Vascular Dementia** |  |  |  |
| VaD as a Proportion of all Dementia | 0.17 | 0.12 | 0.2 |
| **Losses from Morbidity** |  | 95% CI LL | 95% CI UL |
| Average Wage | 17.2742955 | 17.20658154 | 17.3420095 |
| **Inpatient Unit Cost** |  |  |  |
| England | 4130436 | 1103.954671 | 4559814117 |
| Scotland | 144,214 | 3637.779655 | 524618755 |
| Northern Ireland | 53994 | 2393.480952 | 129233611 |
| **Outpatient Unit Costs** |  |  |  |
| England | 5910223.58 | 123.3749198 | 729173360 |
| Scotland | 253245.698 | 164.2873617 | 41605067.6 |
| Northern Ireland | 148383.794 | 161.2775447 | 23930974 |

## Appendix H. Informal Care Cost Variables

|  | Base-Case | N | SE | Distribution |
| --- | --- | --- | --- | --- |
| CVD Prevalence | 0.3223038 | 210,525 | 0.0010186 | Beta |
|  |  |  |  |  |
| Probabilities of: |  |  |  |  |
| Severe Disability | 0.1448117 | 24526 | 0.003754 | Beta |
| Receiving Help (out-of-household) | 0.2426607 | 21216 | 0.0045428 | Beta |
| Receiving Help (in-household) | 0.1123566 | 10017 | 0.0042088 | Beta |
| Needing Helper 1 Daily | 0.1232629 | 7631 | 0.0045403 | Beta |
| Needing Helper 1 Weekly | 0.2812439 | 7631 | 0.0061638 | Beta |
| Needing Helper 1 Monthly | 0.2080903 | 7631 | 0.0048659 | Beta |
| Needing Helper 1 Yearly | 0.3874029 | 7631 | 0.0080886 | Beta |
| Needing Helper 2 Daily | 0.0891688 | 3212 | 0.0056988 | Beta |
| Needing Helper 2 Weekly | 0.3106511 | 3212 | 0.0100046 | Beta |
| Needing Helper 2 Monthly | 0.2524619 | 3212 | 0.0079535 | Beta |
| Needing Helper 2 Yearly | 0.3477182 | 3212 | 0.0117115 | Beta |
| Needing Helper 3 Daily | 0.0817662 | 1226 | 0.008625 | Beta |
| Needing Helper 3 Weekly | 0.3015859 | 1226 | 0.0157771 | Beta |
| Needing Helper 3 Monthly | 0.2808119 | 1226 | 0.0133858 | Beta |
| Needing Helper 3 Yearly | 0.335836 | 1226 | 0.0180879 | Beta |
| Helper 1 Daily Hours | 6.47432 | 8009 | 1.250886 | Gamma |
| Helper 1 Weekly Hours | 4.602524 | 11166 | 0.6529307 | Gamma |
| Helper 1 Monthly Hours | 6.130282 | 7616 | 0.4781576 | Gamma |
| Helper 1 Yearly Hours | 12.28493 | 12760 | 0.8061477 | Gamma |
| Helper 2 Daily Hours | 5.847826 | 2500 | 0.909332 | Gamma |
| Helper 2 Weekly Hours | 5.334342 | 5527 | 0.7889071 | Gamma |
| Helper 2 Monthly Hours | 5.773077 | 4359 | 0.5642227 | Gamma |
| Helper 2 Yearly Hours | 11.35959 | 5327 | 0.9788509 | Gamma |
| Helper 3 Daily Hours | 8.507692 | 812 | 3.050598 | Gamma |
| Helper 3 Weekly Hours | 4.863208 | 2074 | 0.5825691 | Gamma |
| Helper 3 Monthly Hours | 6.431138 | 1818 | 0.9358536 | Gamma |
| Helper 3 Yearly Hours | 11.47674 | 2195 | 1.368099 | Gamma |
| Helper 1 Employed | 0.6941486 |  | 0.0023629 | Beta |
| Helper 1 Unemployed | 0.3058514 |  |  | Beta |
| Helper 2 Employed | 0.7834669 |  | 0.0031383 | Beta |
| Helper 2 Unemployed | 0.2165331 |  |  | Beta |
| Helper 3 Employed | 0.7757134 |  | 0.0050856 | Beta |
| Helper 3 Unemployed | 0.2242866 |  |  | Beta |
|  |  |  |  |  |
| **Wages** |  |  |  |  |
| Minimum Wage (Hourly) | £ 8.21 |  |  |  |
| Average Employed Wage (Hourly) | £ 17.97 | 26,703,000 | 0.05391 | Gamma |
